# A Novel Microglia-Specific Transcriptional Signature Correlates With Behavioral Deficits in Neuropsychiatric Lupus

**DOI:** 10.3389/fimmu.2020.00230

**Published:** 2020-02-26

**Authors:** Hadijat M. Makinde, Deborah R. Winter, Daniele Procissi, Elise V. Mike, Ariel D. Stock, Mary J. Kando, Gaurav T. Gadhvi, Steven Droho, Christina L. Bloomfield, Salina T. Dominguez, Maximilian G. Mayr, Jeremy A. Lavine, Chaim Putterman, Carla M. Cuda

**Affiliations:** ^1^Division of Rheumatology, Department of Medicine, Feinberg School of Medicine, Northwestern University, Chicago, IL, United States; ^2^Department of Radiology, Feinberg School of Medicine, Northwestern University, Chicago, IL, United States; ^3^Division of Rheumatology, Department of Medicine, Albert Einstein College of Medicine, The Bronx, NY, United States; ^4^Department of Physiology, Feinberg School of Medicine, Northwestern University, Chicago, IL, United States; ^5^Department of Ophthalmology, Feinberg School of Medicine, Northwestern University, Chicago, IL, United States; ^6^Research Division, Azrieli Faculty of Medicine and Galilee Medical Center, Nahariya, Israel

**Keywords:** lupus, SLE, NP-SLE, behavior, microglia, DAM, interferon

## Abstract

Neuropsychiatric symptoms of systemic lupus erythematosus (NP-SLE) affect over one-half of SLE patients, yet underlying mechanisms remain largely unknown. We demonstrate that SLE-prone mice (CReCOM) develop NP-SLE, including behavioral deficits prior to systemic autoimmunity, reduced brain volumes, decreased vascular integrity, and brain-infiltrating leukocytes. NP-SLE microglia exhibit numerical expansion, increased synaptic uptake, and a more metabolically active phenotype. Microglia from multiple SLE-prone models express a “NP-SLE signature” unrelated to type I interferon. Rather, the signature is associated with lipid metabolism, scavenger receptor activity and downregulation of inflammatory and chemotaxis processes, suggesting a more regulatory, anti-inflammatory profile. NP-SLE microglia also express genes associated with disease-associated microglia (DAM), a subset of microglia thought to be instrumental in neurodegenerative diseases. Further, expression of “NP-SLE” and “DAM” signatures correlate with the severity of behavioral deficits in young SLE-prone mice prior to overt systemic disease. Our data are the first to demonstrate the predictive value of our newly identified microglia-specific “NP-SLE” and “DAM” signatures as a surrogate for NP-SLE clinical outcomes and suggests that microglia-intrinsic defects precede contributions from systemic SLE for neuropsychiatric manifestations.

## Introduction

Systemic lupus erythematosus (SLE) is a chronic autoimmune disease that arises from the combination of genetic and environmental factors, culminating in destructive tissue injury to multiple organ systems including the central nervous system (CNS). Although incidence varies widely among evaluated cohorts (1), nearly one-half of patients experience primary, or disease-related, manifestations of CNS disease, known as neuropsychiatric SLE (NP-SLE). NP-SLE may be among the earliest signs of SLE (2), but NP-SLE manifestations lack correlation with systemic disease flare (1). As neuropsychiatric symptoms are non-specific and clinically validated biomarkers for diagnosis are nonexistent, primary NP-SLE diagnosis is routinely documented by ruling out secondary causes and in some cases may even go undiagnosed. NP-SLE can be subdivided into focal or diffuse syndromes. Focal NP-SLE presents as focal seizures, strokes, movement disorders and/or migraine or cluster headache and may involve a predominant ischemic-vascular pathway (1). Patients with diffuse NP-SLE present with symptoms including psychosis, mood disorder, cognitive dysfunction, acute confusional states, headaches other than migraine, or cluster headache and/or anxiety disorders (1). Despite the devastating impact of both focal and diffuse NP-SLE on health-related quality of life, underlying disease mechanisms remain largely unknown, often leading to palliative rather than therapeutic protocols (1).

Pathogenic roles for autoantibodies to brain-specific antigens (anti-ribosomal-P, anti-phospholipid, anti-cardiolipin, anti-NMDAR) are suggested to play a role in NP-SLE (1). Blood-brain-barrier disruption, accelerated atherosclerosis, thrombotic vasculopathy and intrathecal inflammatory cytokines, including type I interferon (IFN), are also proposed as potential contributors to NP-SLE (1). However, these clinical outcomes cannot account for the high prevalence and symptom heterogeneity in NP-SLE patients. Microglia, the tissue-resident macrophages of the brain, are gaining more attention in the pathogenesis of NP-SLE (3). Microglia establish permanent residence during embryonic development and serve as first responders to neuronal damage and infections to restore/maintain homeostasis (4). Microglia also play critical roles in shaping neural circuit connectivity and pruning synaptic connections by engulfing pre-/post-synaptic elements (4). Studies show histological evidence of perivascular microglial activation (5, 6) in SLE patients and in SLE mouse models based on positive microglial staining of CD68, Iba-1, or F4/80 antigens (7–9). Serum from SLE patients causes inflammatory changes in cultured mouse microglia (10). More recently, excessive synaptic pruning by microglia is associated with behavioral deficits in the 564Igi model of SLE (11), and total macrophage and microglia depletion ameliorates disease in multiple models of NP-SLE (12, 13).

We published that CReCOM mice develop SLE-like disease including splenomegaly, lymphadenopathy, hypergammaglobulinemia, auto-antibody induction (including anti-dsDNA), glomerulonephritis, immune complex deposition in the kidney, exacerbated proteinuria, heightened serum pro-inflammatory cytokines, and increased mortality (14). Here we demonstrate that CReCOM mice also display NP-SLE-like disease. CReCOM mice develop behavioral deficits corresponding to hippocampal and cerebellar defects that precede end-organ pathology, similar to patients with NP-SLE. Magnetic resonance imaging (MRI) shows diminished white matter integrity, decreased vascular integrity, and brain volume reduction in CReCOM mice, consistent with findings among patients (15, 16). CReCOM mice display diffuse leukocyte infiltration, including macrophages, within the brain parenchyma, but not within the choroid plexus. Transcriptional profiling reveals that microglia are more metabolically active in CReCOM mice compared to WT mice. Further, a 18-gene “NP-SLE signature” is shared between microglia of CReCOM mice and the recently validated B6.*Sle1Sle3* NP-SLE model (17). This “NP-SLE signature” is enriched for genes associated with processes related to lipid metabolism, scavenger receptor activity, and downregulating inflammatory responses and cell chemotaxis. NP-SLE microglia are also enriched for genes associated with disease-associated microglia (DAM) observed in multiple neurodegenerative diseases (18). Moreover, expression of “NP-SLE” and “DAM” signatures in microglia correlates with the severity of behavioral deficits prior to overt systemic disease in young SLE-prone mice. These data are the first to connect microglia-specific transcriptional signatures with clinical outcomes in NP-SLE-like disease and suggest that microglia-intrinsic defects precede contributions from systemic SLE for neuropsychiatric manifestations.

## Results

### Behavioral Deficits Precede End-Organ Disease in CReCOM Mice

Since CReCOM mice develop SLE-like disease with age and do not display kidney pathology until 8 months of age (14), we determined whether these mice exhibit NP-SLE-like disease prior to end-organ pathology. To accomplish this, 3–4-month-old female WT and CReCOM, as well as MRL^lpr/lpr^ (positive control), mice, underwent a battery of behavioral tasks validated by Northwestern University's Behavioral Phenotyping Core.

**Figure 1:**
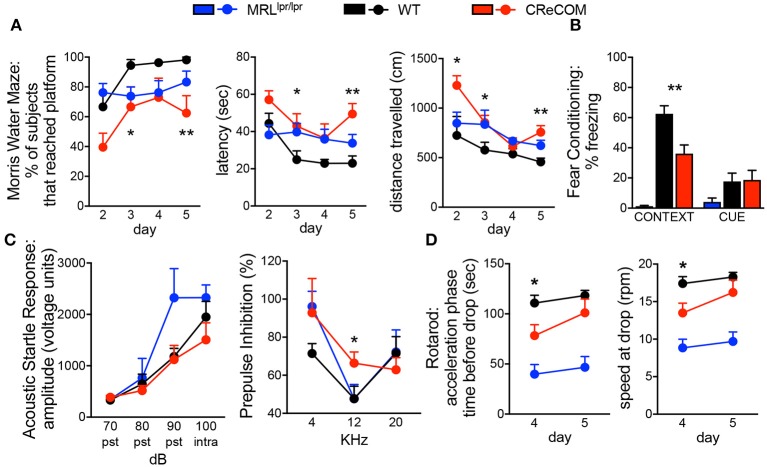
Behavioral deficits occur in young CReCOM mice. 3-4-month-old female MRL^lpr/lpr^ (*n* = 7), WT (*n* = 9), and CReCOM (*n* = 8) mice underwent behavioral testing. Data are combined from 3 independent experiments. **(A)** Morris water maze: % of mice that reached the hidden platform, latency to platform, and distance traveled. **(B)** Fear conditioning: % freezing following reintroduction of “shock” environment (CONTEXT) or tone associated with “shock” (CUE). **(C)** Prepulse inhibition: amplitude of acoustic startle response at 70, 80, 90, and 100 dB and % prepulse inhibition at 4, 12, and 20 KHz frequencies. **(D)** Rotarod: time spent on rod prior to drop and speed at drop during acceleration phase. (*p < 0.05; **p < 0.005).

The Morris water maze assesses hippocampal-dependent spatial memory and learning by testing the ability of animals to remember the location of, and perform the task of climbing onto, a platform in a pool. CReCOM mice exhibited greater latency and traveled greater distances to reach the platform resulting in fewer CReCOM mice reaching the platform than WT mice (Figure 1A). Fear conditioning was also measured to test hippocampal- and/or amygdala-dependent associative learning. CReCOM mice showed less freezing in response to the environment than WT mice (Figure 1B), but showed no defect in response to the cue, indicating a strictly contextual associative learning defect. Prepulse inhibition (PPI) is a measure of CNS activity wherein responses to stronger stimuli are inhibited/dampened by pre-exposure to weaker stimuli (prepulse) and involves the hippocampus, striatum, and brainstem. Acoustic startle response values were similar between CReCOM and WT mice (Figure 1C), indicating normal hearing function. At 4 and 20 KHz prepulse frequencies, CReCOM mice responded similarly to WT mice and confirmed intact hearing function in CReCOM mice. However, CReCOM mice showed a higher %PPI at the 12 KHz prepulse frequency compared to WT mice (Figure 1C), indicating an inability to adapt to the acoustic stimuli even when preceded by a weaker signal. Rotarod evaluates grip strength and motor skill; animals with unimpaired motor coordination will stay on the rod longer than animals with defects in their motor cortex and cerebellum. CReCOM mice were unable to hold on as long and fell off at lower speeds compared to WT mice during the acceleration phase on the first day but were able remain on the rod longer the second day (Figure 1D). Mice were also subjected to zero maze, Y maze, and open field tasks, and data from these tests were similar between WT and CReCOM mice (Supplemental Figures 1A–C). CReCOM mice did not exhibit aberrant gait symmetry or coordination compared to WT mice (Supplemental Figure 1D), signifying the absence of locomotive deficits.

We also subjected 9–10-month-old mice to aforementioned behavioral tasks and found trends toward similar responses as 3–4-month-old CReCOM mice in the Morris water maze, PPI, and Rotarod, but not fear conditioning, tasks compared to WT mice (Supplemental Figures 2A–D); however, aging affects the ability of WT mice to perform tasks (19). Consistent with 3–4-month-old CReCOM mice, 9–10-month-old CReCOM mice behaved similarly to WT mice in the zero maze, Y maze, open field, and DigiGait tasks. We investigated retinal vasculature of 9–10-month-old mice by fluorescein angiography, as retinopathy is a manifestation of SLE (20). We found no irregularities in the retinal vasculature (Supplemental Figure 3), suggesting that behavioral deficits in CReCOM mice were not related to visual deficits. These data confirm spatial memory, contextual associative learning, startle response, and motor coordination defects involving the hippocampus, amygdala, striatum, brainstem, motor cortex, and/or cerebellum regions and indicative of NP-SLE in CReCOM mice prior to end-organ pathology.

### Reduced Brain Volumes, Decreased Connectivity, and Diminished Vascular Integrity in CReCOM Mice

**Figure 2:**
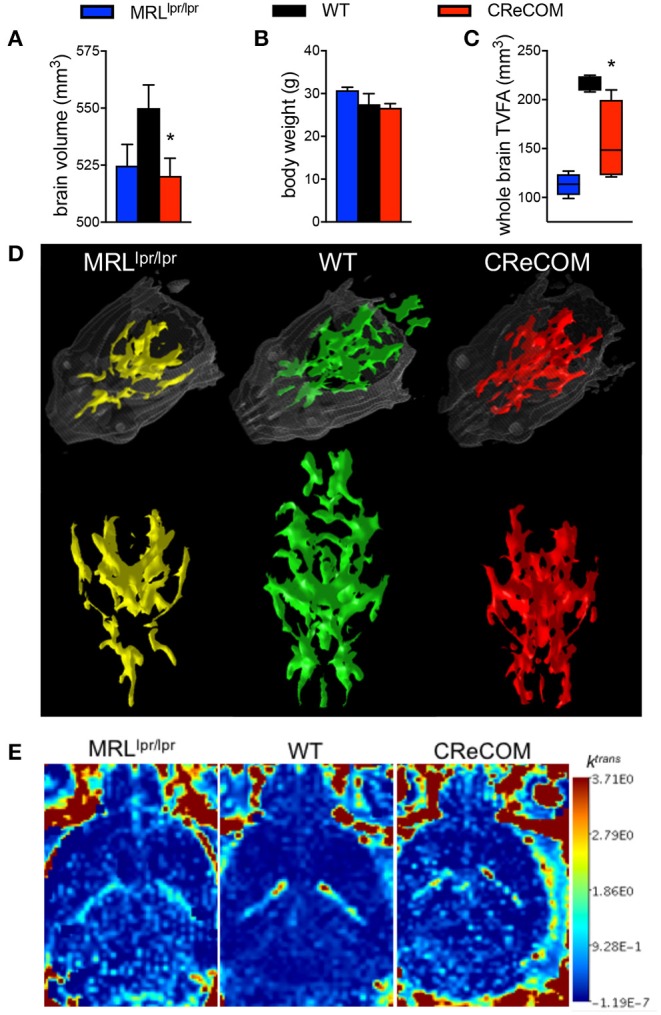
CReCOM mice exhibit reduced brain volumes, decreased connectivity, and diminished vascular integrity. Four-month-old female MRL^lpr/lpr^ (*n* = 4) and 12-month-old female WT (*n* = 4) and CReCOM (*n* = 4) mice underwent MRI. **(A)** Brain volumes extracted from 3D MRI. **(B)** Body weight. **(C)** Fractional anisotropy (FA) maps extracted from diffusion MRI images. A set threshold value (FA ~ 400) generated semi-automated ROI. Volumes of voxels/region with FA values>~400 were determined and thresholded volumetric FA (TVFA) values are shown. **(D)** TVFA renderings overlaid on wired mouse head renderings. **(E)** Images of k^trans^ level (measure of vascular integrity) extracted from dynamic contrast-enhanced MRI (*p < 0.05).

Imaging studies indicate hypermetabolism, corresponding to atrophy, of white and gray brain matter of NP-SLE patients (15, 16). CReCOM mice underwent 3D, diffusion, and dynamic contrast-enhanced MRI to address whether similar radiological findings are evident. CReCOM mice had reduced brain volumes compared to WT mice (Figure 2A), as in NP-SLE patients (15, 16), which were not correlated with decreased body weights (Figure 2B). CReCOM mice exhibited diminished white matter integrity detected via reduced thresholded volumetric fractional anisotropy (TVFA) values compared to WT mice (Figures 2C,D). CReCOM mice also displayed decreased vascular integrity compared to WT mice by intravenous administration of MRI contrast agent, potentially indicating a more permeable blood-brain-barrier similar to NP-SLE patients (1) (Figure 2E). Dynamic contrast-enhanced MRI also showed evidence of a lesion present in one CReCOM brain, but not in WT mice (Supplemental Figure 4). These data indicate that the radiological presentation by CReCOM mice mimics that of NP-SLE patients.

### Evidence of Diffuse Infiltration in Brains of CReCOM Mice

Previous data suggest that leukocytes infiltrate brains of NP-SLE patients and models of NP-SLE (5, 21–25). We observed evidence of diffuse CD45 expression within the cerebellum, but not the choroid plexus, of CReCOM mice compared to WT mice by histology (Supplemental Figure 5). We then confirmed histological data using flow cytometric analysis.

**Figure 3:**
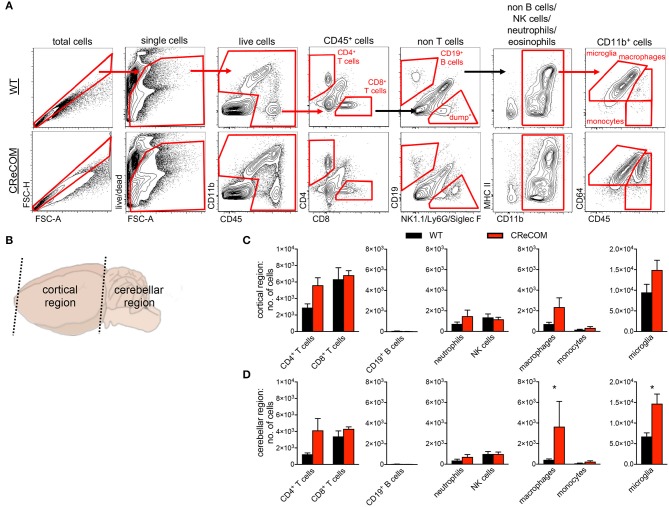
Evidence of diffuse leukocyte infiltration in CReCOM brains. Brains of 11-12-month-old female WT (*n* = 4) and CReCOM (*n* = 4) mice were split into two portions and analyzed by flow cytometry. Data are representative of three independent experiments. **(A)** Whole brain gating strategy. **(B)** Regional breakdown for subsequent analysis. **(C)** Analysis of cortical region infiltration of T-cells (CD4^+^CD45^hi^ and CD8^+^CD45^hi^), B-cells (CD19^+^CD45^hi^), neutrophils (CD11b^+^Ly6G^+^CD45^hi^), NK cells (CD11b^+^NK1.1^+^CD45^hi^), macrophages (CD11b^+^CD64^+^CD45^hi^), monocytes (CD11b^+^CD64^−^CD45^hi^), as well as microglia (CD11b^+^CD64^+^CD45^lo^). **(D)** Analysis of aforementioned populations in cerebellar region (*p < 0.05).

We regionally examined the brain: choroid plexus alone (from all ventricles), cortical region with corresponding choroid plexus, and cerebellar region with corresponding choroid plexus (Supplemental Figure 6 and Figure 3). We did not find leukocyte infiltration within the choroid plexus of CReCOM mice (Supplemental Figures 6A,B), similar to our histological observations (Supplemental Figure 5). However, the cortical region of CReCOM brains showed a trend toward increased macrophage infiltration, as well as elevated numbers of microglia, compared to WT brains (Figures 3A–C). Further, significant infiltration of macrophages and expansion of microglia was observed in cerebellar region of CReCOM mice compared to WT mice (Figures 3A,B,D). Cortical and cerebellar regions of CReCOM brains showed increased CD4^+^ T-cell infiltration compared to WT brains (Figures 3C,D). Our histological and flow cytometric data confirmed massive infiltration within the choroid plexus of MRL^lpr/lpr^ brains (Supplemental Figures 5, 6A,B). Thus, CReCOM mice exhibit diffuse leukocyte infiltration into the brain parenchyma, potentially owing to decreased vascular integrity, similar to NP-SLE patients.

### Disease-Specific Alterations in Gene Expression of CReCOM Macrophages and Microglia

**Figure 4:**
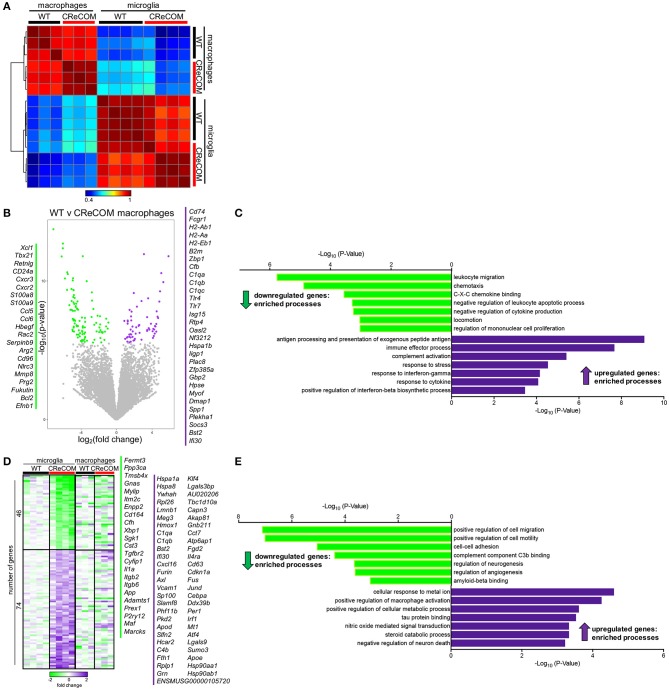
Microglia and infiltrating macrophages in CReCOM brains show distinct transcriptional profiles. FACS-purified macrophages of 11-12-month-old female WT (*n* = 3) and CReCOM (*n* = 3) mice and microglia of 11-12-month-old female WT (*n* = 4) and CReCOM (*n* = 4) mice were analyzed by RNA-seq. **(A)** Pairwise Pearson correlation of gene expression between macrophage and microglia samples from CReCOM and WT mice. **(B)** Volcano plot depicting the most differentially expressed genes (167) (DESeq2, false discovery rate (FDR) <10%, fold change>1.5) between WT and CReCOM macrophages. Downregulated genes (105) in CReCOM shown in green; upregulated genes (62) in CreCOM shown in purple. **(C)** GO processes associated with differentially expressed genes in CReCOM macrophages. **(D)** Heat map depicting relative expression [log_2_[fold change compared to mean of WT replicates]] of the most differentially expressed genes (120) (DESeq2, FDR <10%, fold change>1.5) between WT and CReCOM microglia. Downregulated genes (46) in CReCOM shown in green; upregulated genes (74) in CReCOM shown in purple. Macrophage expression data for microglia-specific differentially expressed genes are depicted. **(E)** GO processes associated with differentially expressed genes in CReCOM microglia.

We previously showed that preventing monocyte-derived macrophage infiltration into the brain improved behavioral deficits and white matter integrity in a traumatic brain injury model (26, 27). Since CReCOM mice showed behavioral deficits corresponding to macrophage infiltration as well as increased numbers of microglia, we determined gene expression profiles of fluorescence-activated cell sorting (FACS)-purified macrophages and microglia from WT and CReCOM mice by RNA-seq. Isolated populations were transcriptionally distinct (Figure 4A). A number of genes are enriched in microglia compared to other myeloid and CNS populations (11, 28), and we compared expression between isolated macrophages and microglia (Supplemental Figure 7A). Of the 47 genes evaluated, 39 genes were expressed at higher levels in microglia than in macrophages (*Cx3cr1*:12-fold, *P2ry12*:34-fold, *P2ry13*:77-fold, *Tmem119*:41-fold, *Trem2*:14-fold, *Olfml3*:24-fold, *Gpr34*:44-fold). The remaining genes were either expressed at higher levels in macrophages than in microglia or were not detected in either population (Supplemental Figure 7A). These data demonstrate distinct macrophage and microglia populations by transcriptional profiling (Supplemental Figure 7A) and cell surface expression of known antigens (Figure 3A).

To evaluate the pathways altered in CReCOM macrophages, the most differentially expressed genes between WT and CReCOM microglia were determined (Figure 4B). Macrophages from CReCOM mice downregulated 105 genes (*S100a8, S100a9, Cxcr2, Ccl6, Bcl2*) related to *leukocyte migration, chemotaxis, C-X-C chemokine binding, negative regulation of leukocyte apoptotic process, negative regulation of cytokine production, locomotion*, and *regulation of mononuclear cell proliferation* compared to WT mice (Figure 4C). These macrophages also upregulated 62 genes (*Tlr4, Tlr7, CD74, Bst2, Ifi30*) associated with *antigen processing and presentation of exogenous peptide antigen, immune effector process, complement activation, response to stress, response to interferon-gamma, response to cytokine*, and *positive regulation of interferon-beta biosynthetic process* (Figure 4C). These data indicate that infiltrating CReCOM macrophages may be capable of increased antigen presentation and complement activation, as well as be more responsive to IFN present in the environmental milieu.

We next evaluated transcriptional pathways altered in CReCOM microglia. The most differentially expressed genes between WT and CReCOM microglia were determined (Figure 4D). Microglia were depleted for 46 genes (*Cd164, Sgk1, Il1a, Maf* , *Marcks*) related to *positive regulation of cell migration, positive regulation of cell motility, cell-cell adhesion, complement component C3b binding, regulation of neurogenesis, regulation of angiogenesis*, and *amyloid beta binding* processes in CReCOM compared to WT mice (Figure 4E). On the other hand, microglia were enriched for 74 genes (*Klf4, Jund, Cd63, Sumo3, Atf4*) associated with *cellular response to metal ion, positive regulation of macrophage activation, positive regulation of cellular protein metabolic process, tau protein binding, nitric oxide mediated signal transduction, steroid catabolic process*, and *negative regulation of neuron death* processes in CReCOM compared to WT mice (Figure 4E). This expression profile is consistent with a metabolically active microglia phenotype in CReCOM mice.

We then evaluated the pattern of expression of microglia-associated differentially expressed genes in brain-infiltrating macrophages to determine whether a “SLE signature” is present in these populations of CReCOM mice (Figure 4E). We did not observe a similar pattern of expression of microglia-associated differential genes (Figure 4E), suggesting that brain-infiltrating macrophages and microglia of CReCOM mice do not share a common signature. Since prior work suggested an IFN signature was present in microglia from the 564Igi NP-SLE model (11), we examined expression of IFN response genes upregulated in 564Igi microglia in both CReCOM microglia and macrophages. CReCOM microglia did not show increased expression of IFN response genes, unlike 564Igi microglia (Supplemental Figures 7B,C). However, CReCOM macrophages were not only enriched for genes associated with *response to interferon-gamma* (*H2-Ab1, H2-Aa, Bst2, Tlr4, Gbp2, H2-Eb1*) and *positive regulation of interferon-beta biosynthetic process* (*Tlr4, Tlr7*) processes (Figures 4B,C), but also showed increased expression of IFN response genes observed in 564Igi microglia (Supplemental Figure 7B). Breakdown of these IFN response genes indicated that only 25% were increased in CReCOM microglia (75% were either not expressed, not different from WT, or decreased compared to WT), while 75% of genes were elevated in CReCOM macrophages (Supplemental Figure 7C). These data suggest that infiltrating macrophages and microglia possess distinct functions in NP-SLE-like disease.

### Increased Activation of CReCOM Microglia

Previous studies show histological evidence of microglial activation in NP-SLE models via CD68 staining (7–9). Further, microglia from 564Igi mice showed increased IFNAR-dependent internalization of a synaptic vesicle protein, suggesting enhanced engulfment of synaptic material (11). To determine whether microglia of CReCOM mice exhibit functional defects, we examined their activation status and internalization of synaptic material via flow cytometric analysis. Activated CD68^+^ microglia were increased in CReCOM compared to WT mice (Supplemental Figures 8A,B). Further, CReCOM microglia showed elevated uptake of synaptic material, via intracellular staining of synaptic vesicle protein SV2A, compared to WT microglia (Supplemental Figures 8C,D). Thus, intrinsically defective CReCOM microglia may be more functionally active than WT microglia due to IFN-independent mechanisms.

### A Shared Microglia-Specific Transcriptional Signature in CReCOM and B6.*Sle1Sle3* Mice

**Figure 5:**
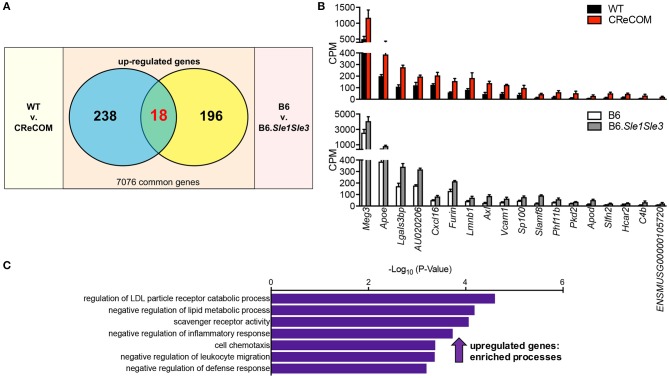
Evidence of a common “NP-SLE signature” in microglia. FACS-purified microglia of 11-12-month-old female WT (*n* = 4), and CReCOM (*n* = 4) mice were analyzed by RNA-seq and compared to data from 8-10-month-old female B6 and B6.*Sle1Sle3* mice (17). **(A)** A significant overlap exists between upregulated genes (DESeq2, *p* < 0.05, fold change>1.5) in CReCOM (256) and B6.*Sle1Sle3* (214) microglia (*p* < 2.54 × 10^−4^, hypergeometric distribution). **(B)** Expression values (counts per million=CPM) for shared genes. **(C)** GO processes associated with shared genes.

We recently published that the SLE-prone B6.*Sle1Sle3* strain also functions as a NP-SLE model (17). Further, we showed that microglia from these mice present an altered transcriptional profile (17). To determine whether a shared disease signature exists between CReCOM and B6.*Sle1Sle3* microglia, we compared differentially expressed genes between CReCOM and WT microglia with those between B6.*Sle1Sle3* and B6 microglia. Of the genes upregulated in CReCOM (256) and B6.*Sle1Sle3* (214) microglia compared their respective controls, a significant number were shared (Figures 5A,B). This “NP-SLE signature” was enriched for genes involved in *regulation of LDL particle receptor catabolic process, negative regulation of lipid metabolic process, scavenger receptor activity, negative regulation of inflammatory response, cell chemotaxis, negative regulation of leukocyte migration*, and *negative regulation of defense response* processes (Figure 5C). Together, these data suggest that microglia may not be adequately functioning to suppress inflammation in the brain and that a common “NP-SLE signature” may be penetrant in microglia across multiple NP-SLE models.

### Expression of DAM-Associated Genes in CReCOM and B6.*Sle1Sle3* Microglia

**Figure 6:**
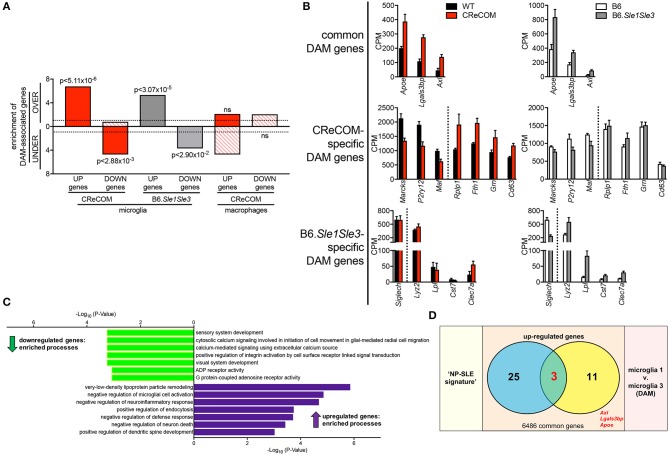
Expression of DAM-associated genes in NP-SLE microglia. FACS-purified microglia of 11-12-month-old female WT (*n* = 4), and CReCOM (*n* = 4) mice were analyzed by RNA-seq and compared to data from 8-10-month-old female B6 and B6.*Sle1Sle3* mice (17). Differential genes (DESeq2, *p* < 0.05, fold change>1.5) in CReCOM (581) and B6.*Sle1Sle3* (472) microglia compared to their respective controls were determined. Differential genes (fold change in average UMI count>1.5) between Microglia3 (DAM) and Microglia1 populations (48) were determined (29). **(A)** Enrichment of significantly upregulated (UP) and downregulated (DOWN) genes in microglia or macrophages from NP-SLE models in the over-(OVER) or under-(UNDER)-expressed genes in DAMs. Enrichment=ratio of the observed number of genes that overlap DAM-associated genes divided by the expected number. Dotted line at enrichment value of “1” denotes observed number equals expected number of overlapping genes due to chance. *P*-values reflect hypergeometric distribution. **(B)** Expression values (CPM) for shared genes in microglia from NP-SLE-like disease models and DAM, termed “DAM signature.” **(C)** GO processes associated with 15-gene “DAM signature.” **(D)** Overlapping genes between upregulated genes in “NP-SLE” and “DAM” signatures (*p* < 2.70 × 10^−7^, hypergeometric distribution).

DAM were recently identified in mouse models of neurodegenerative diseases, including Alzheimer's disease (AD), amyotrophic lateral sclerosis (ALS), and multiple sclerosis (MS) and verified in human disease (18). We compared the DAM profile to microglia profiles of CReCOM and B6.*Sle1Sle3* mice. We identified significant overlap between upregulated and downregulated genes of CReCOM and B6.*Sle1Sle3* mice with DAM counterparts (29) (Figure 6A). We examined CPM levels of all DAM-associated genes found to be differentially expressed in microglia of either CReCOM or B6.*Sle1Sle3* mice compared to their respective controls, termed “DAM signature”. We found similar trends in expression of CReCOM-specific DAM genes (*Marcks, P2ry12, Maf* , *Rplp1, Fth1*) in B6.*Sle1Sle3* microglia and of B6.*Sle1Sle3*-specific DAM genes (*Lyz2, Clec7a*) in CReCOM microglia (Figure 6B). The “DAM signature” was depleted for genes related to *sensory system development, cytosolic calcium signaling involved in initiation of cell movement in glial-mediated radial cell migration, calcium-mediated signaling using extracellular calcium source, positive regulation of integrin activation by cell surface receptor linked signal transduction, visual system development, ADP receptor activity*, and *G protein-coupled adenosine receptor activity* processes (Figure 6C). The “DAM signature” was enriched for genes associated with *very-low-density lipoprotein particle remodeling, negative regulation of microglial cell activation, negative regulation of neuroinflammatory response, positive regulation of endocytosis, negative regulation of defense response, negative regulation of neuron death*, and *positive regulation of dendritic spine development* processes (Figure 6C). Further, we found significant overlap of three genes (*Apoe, Axl, Lgals3bp*) in “NP-SLE” and “DAM” signatures (Figure 6D). There was no overlap between the “DAM signature” and the CReCOM macrophage profile (Figure 6A). These data suggest that microglia in NP-SLE-like disease may be enriched for DAM.

### Expression of the “NP-SLE Signature” Correlates With Behavioral Deficits in CReCOM Mice

**Figure 7:**
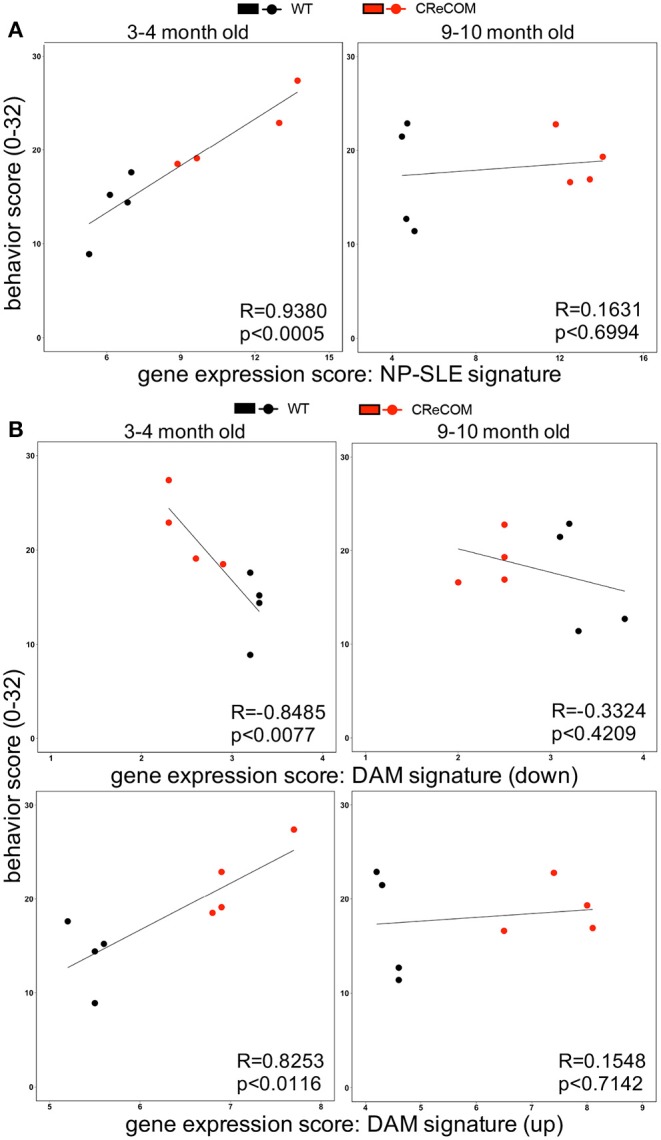
Expression of “NP-SLE” and “DAM” signatures correlates with behavioral deficits in young CReCOM mice. FACS-purified microglia of 5-6-month-old and 11-12-month-old female WT (*n* = 4) and CReCOM mice (*n* = 4) mice were analyzed by RNA-seq. Correlation coefficients (R) were determined between behavior (y-axis) and gene expression (x-axis) scores. Lines suggest pattern of correlation. Scatter plots depicting correlation between **(A)** “NP-SLE signature” or **(B)** downregulated and upregulated “DAM signature” gene expression scores and behavior scores.

Our data suggest that microglia from 8–12-month-old mice exhibiting NP-SLE-like disease share a “NP-SLE signature” and are enriched for DAM-associated genes. Thus, we determined whether expression of these signatures correlates with behavioral deficits in CReCOM mice. We generated a behavior score and gene expression scores for “NP-SLE” and “DAM” signatures for each mouse from both 3–4-month-old (corresponding to 5–6-month-old RNA-seq data) and 9–10-month-old (corresponding to 11–12-month-old RNA-seq data) cohorts. Microglia from the 3–4-month-old cohort showed a similar expression pattern of most genes associated with “NP-SLE” and “DAM” signatures (Supplemental Figure 9). A strong correlation was observed between behavior and the “NP-SLE signature” (Figure 7A), as well as both the downregulated and upregulated “DAM signature” gene expression scores (Figure 7B), in the 3–4-month-old cohort. No correlations were found between behavior and gene expression scores in the 9–10-month-old cohort (Figures 7A,B), most likely due to the fact that behavioral data from this cohort reflects deficits associated with aging in WT mice (19). These data suggest that “NP-SLE” and the “DAM” signatures observed in 8–12-month-old mice are predictive of behavioral deficit severity earlier in NP-SLE-like disease.

## Discussion

Here, for the first time, we uncover a shared “NP-SLE signature” and enrichment of DAM-associated genes in microglia from multiple NP-SLE models. We find that NP-SLE-like disease in CReCOM mice mimics aspects of NP-SLE patient presentation, including behavioral deficits corresponding to hippocampal and cerebellar (30–32) defects that precede end-organ pathology, diminished white matter integrity, decreased vascular integrity and brain volume reduction, as well as lymphocytic and macrophage infiltration within the brain (1, 5, 16, 21–23, 25, 30–32). Moreover, this cellular infiltrate is diffuse within the brain parenchyma of CReCOM mice and not localized to the choroid plexus. Transcriptional profiling of brain-infiltrating macrophages and microglia of CReCOM mice shows distinct, non-overlapping signatures. CReCOM macrophages are enriched for genes associated with increased antigen presentation and complement activation, as well as an IFN signature. In contrast, CReCOM microglia do not exhibit an IFN signature and are enriched for genes indicating a more metabolically active phenotype. Comparison of transcriptional profiles between CReCOM and B6.*Sle1Sle3* microglia reveals a shared “NP-SLE signature,” and both CReCOM and B6.*Sle1Sle3* microglia are enriched for DAM-associated genes. More importantly, expression of genes linked to “NP-SLE” and “DAM” signatures correlates with clinical outcomes in CReCOM mice.

A recent study reveals that microglia exhibit enhanced synaptic pruning leading to synapse loss and development of NP-SLE-like disease using the B-cell transgenic 564Igi SLE model (11). This strain displays anxiety, cognitive defects, increased social aggression, abnormal social interaction, and enhanced PPI, and the authors suggest that microglia-dependent synapse loss mediates these neurological symptoms (11). Similarly, spatial memory impairment in DNRb+ mice is facilitated by microglial-mediated C1q-dependent neuronal damage (13). We also find that CReCOM microglia show increased uptake of synaptic material, implying excessive synaptic pruning by this population. As in the 564Igi (11) and DNRAb+ (13) models, CReCOM mice present increased CD68^+^ reactive microglia. Both the IFNα receptor 1 (IFNAR)-dependent systemic autoimmunity (33) and NP-SLE-like disease in the 564Igi model require peripheral cells to produce type I IFN, which cross the blood-brain-barrier to activate microglia, induce microglia-dependent synapse loss, and cause presentation of an IFN signature in microglia (11). Similar to patients with NP-SLE (5, 21–23), CReCOM mice display diffuse infiltration of T-cells and macrophages. However, in 564Igi mice, there are no brain-infiltrating immune cells, and follicular DCs but not plasmacytoid DCs produce type I IFN (34), both of which are inconsistent with human pathology (5, 21–23). The requirement for IFN-mediated synaptic pruning in 564Igi mice was confirmed using head-shielded IFNAR^−/−^ bone marrow chimeric mice (11). However, since all cells in the chimeric mice, including astrocytes, neurons, and microglia, lack IFNAR, a direct role for microglia cannot be ascertained. A recent study showed that type I IFN binds to neurons or astrocytes to activate microglia in a paracrine fashion (35). Further, previous studies suggest that type I IFN weakly pass through the blood-brain-barrier and may also be protective (36–38), which only complicates the role that type I IFN plays in NP-SLE. In contrast to these findings, microglia from CReCOM and B6.*Sle1Sle3* mice do not exhibit an IFN signature. *Ifnb* and several genes induced by IFN (*Isg15, Ifit1, Trim12a*) are actually downregulated by at least 2-fold in B6.*Sle1Sle3* microglia compared with B6 microglia (17). Rather, in CReCOM mice, it is the brain-infiltrating macrophages that exhibit an IFN signature. Thus, despite detection of circulating IFNα and IFNβ in CReCOM mice (14) and IFNα in B6.*Sle1Sle3* mice (39), these cytokines do not seem to induce an IFN signature in microglia of these models. While 50–80% of patients display an IFN signature (40), 20–50% do not. As it is unclear whether IFNAR signaling is increased in brains of SLE patients and/or linked to NP-SLE, perhaps circulating IFN may not affect microglia in all NP-SLE patients.

Infiltrating immune cells have recently emerged as an important component of the disease-associated microenvironment in the brain and may participate in neurodegenerative disease progression (41). Since monocyte-derived macrophages play role in SLE-like disease (42–44), we cannot rule out that brain-infiltrating macrophages may contribute to NP-SLE-like disease. CReCOM mice exhibit infiltration of macrophages into the brain parenchyma that do not show caspase-8 deletion. However, these macrophages, though not intrinsically altered by the flox-cre construct, are enriched for genes associated with increased antigen presentation and complement activation, as well as an IFN signature. We previously showed that preventing monocyte-derived macrophage infiltration into the brain improved behavioral deficits and white matter integrity in a traumatic brain injury model (26, 27). Thus, our data do not preclude a contributing role for monocyte-derived macrophages in the development of NP-SLE-like disease in CReCOM mice, as previous studies suggest that infiltrating macrophages may modulate the activity of microglia (45, 46). Global depletion of monocyte-derived macrophages and tissue-resident macrophages, including microglia, via inhibition of colony-stimulating factor-1 receptor signaling which is crucial for their development, improves NP-SLE-like disease in MRL^lpr/lpr^ and DNRAb+ mice (12, 13). We recently demonstrated that restoration of the blood-brain-barrier in MRL^lpr/lpr^ mice reduces systemic autoimmunity and NP-SLE-like disease without affecting pro-inflammatory cytokines or autoantibodies but decreases the numbers of infiltrating monocyte-derived macrophages into the choroid plexus (47). However, it is possible that the blood-cerebrospinal fluid-barrier (choroid plexus) may be more important for immune cell trafficking in the MRL^lpr/lpr^ model than other models of NP-SLE (48), including CReCOM and B6.*Sle1Sle3* (17) mice. NP-SLE-like disease persists in MRL^lpr/lpr^ mice transferred with healthy bone marrow despite mitigation of systemic disease (24), suggesting a brain-intrinsic mechanism rather than peripheral hematopoietic cell contribution for NP-SLE development. Similar to these findings in MRL^lpr/lpr^ mice, the lack of systemic disease attenuation following deletion of LCN2 in B6.*Sle1Sle3* mice suggests that the corrective effects on NP-SLE seen by LCN2 deficiency are primarily the result of brain-specific pathways (17) rather than decreased systemic inflammation or brain-infiltrating T-cells or macrophages. However, we cannot rule out the effect of increased levels of brain-infiltrating CD4^+^ T-cells on microglia activation in NP-SLE-like disease (49). Recent studies suggest that loss of CD4^+^ T-cells reduces dopaminergic cell death and microglia activation in a mouse model of Parkinson's disease, a chronic neurodegenerative condition (50). Thus, it could be argued that increasing CD4^+^ T-cell infiltration into the brains of CReCOM and B6.*Sle1Sle3* mice (17) may contribute to microglia activation and thus to NP-SLE-like disease itself and merits further investigation. These data suggest that microglia are critical for NP-SLE-like disease, but brain-infiltrating macrophages, and possibly CD4^+^ T-cells, may synergize with microglia to initiate and perpetuate cognitive decline. Though we do not focus on non-immune cells, we cannot rule out the effect of astrocyte-mediated control of microglia activation, phagocytic capacity, and ability to secrete inflammatory mediators via cell-cell interaction (51) in NP-SLE pathogenesis and merits further investigation.

We recently demonstrated that the B6.*Sle1Sle3* SLE model also develops NP-SLE-like disease (17). Similar to CReCOM mice, B6.*Sle1Sle3* mice show a coordination defect (17). Impaired working memory and compromised spatial and recognition memory are seen in CReCOM and B6.*Sle1Sle3* mice (17), respectively. Further, similar to CReCOM mice, B6.*Sle1Sle3* mice show diffuse infiltration of T-cells and macrophages within the brain parenchyma rather than the choroid plexus (17). The CReCOM and B6.*Sle1Sle3* strains share a common B6 background, which allows for comparisons of cell-specific transcriptional profiles without interference of unknown disease mechanisms specific to a different background. Comparison of CReCOM and B6.*Sle1Sle3* microglial transcriptional profiles yields a shared 18-gene signature that appears to indicate a more anti-inflammatory, regulatory phenotype in microglia in the later stages of disease. Thus, despite these strains being generated and housed in different facilities (microbiome, diet, care) and the fact that the mechanisms of systemic disease differ (cell-specific caspase-8 deficiency vs. *Sle* susceptibility loci), a common “NP-SLE-disease signature” exists within microglia. Our results identify multi-gene signatures that are consistent across microglia of two NP-SLE models and observed in published microglia datasets rather than define a single-gene/pathway as a contributor to disease; thus, target validation was not necessitated. Further, we find significance not only in differential expression of our signatures but also in correlation of signature expression to clinical outcomes, indicating a sufficient sample size. Future studies beyond the scope of this study include the use of spatial transcriptomics to verify microglia gene signatures from whole brain in specific regions.

A number of genes comprising the “NP-SLE signature” are highly relevant to microglial function [*Furin* (52), *Vcam-1* (53), *Cxcl16* (54), *C4b* (55)], aging [*Sp100* (56)], and activation [*ApoD* (57)]. Numerous genes have also been implicated in neurodegenerative disease [*Vcam-1* (53), *Lmnb1* (58)*, Hcar2* (59), *ApoE* (60), *Axl* (61, 62)]. Expression of *Apoe* is upregulated in late-response state microglia, along with *Cxcl16, C4b, Lgals3bp*, and *Axl*, in an inducible model of severe neurodegeneration (63) and mediates the transition from the homeostatic to DAM phenotype in microglia (64). Further, expression of *ApoE, Axl*, and *Lgals3bp* is shared between microglia from both NP-SLE models and DAM. Interestingly, genes not typically associated with microglia were upregulated in NP-SLE populations. Slamf8 negatively regulates NOX2 activity in macrophages in response to multiple stimuli (65). *Meg3* is a repressive chromatin-interacting long non-coding RNA that directly targets TGF-β pathway genes to regulate signaling (66). Polycystin-2 (encoded by *Pkd2*) functions as an ion channel permeable for calcium ions, and its overexpression leads to augmented intracellular calcium release signals in epithelial cells (67). *PHF11* (human ortholog of *Phf11b*) RNA, as well as nuclear localization of PHF11, increases in TLR3-activated keratinocytes (68) and activated T-cells (69). Schlafen 2 (encoded by *Slfn2*) interacts with PPP6R1, leading to decreased type 1 IFN-induced activation of NF-κB signaling, causing a reduction of IFN-stimulated genes in mouse embryonic fibroblasts (70), suggesting Slfn2 negatively regulates IFN-stimulated gene expression. In addition, increased levels of proteins encoded by several genes in our “NP-SLE signature” are linked to active renal disease in SLE [*Cxcl16* (71, 72), *Axl* (73, 74), *Lgals3bp* (75), *Vcam-1* (72), *ApoE* (76)]. Moreover, Vcam-1 levels correlate with cognitive brain mechanisms in SLE patients (77) and the *APOE*ε4 allele is reported to be associated with NP-SLE in SLE patients (78). Furthermore, increasing expression of all aforementioned genes in CReCOM microglia in the earlier stages of SLE- and NP-SLE-like disease correlates with behavioral deficit severity, potentially suggesting a microglia-mediated brain-intrinsic mechanism for onset of NP-SLE manifestations.

A TREM2-dependent microglial subset that is enriched for lipid metabolism pathways and phagocytosis-related functions during disease was recently identified by single-cell RNA-seq. This microglial subset is referred to as disease-associated microglia (DAM), and its presence has been verified in human disease (18). Previous studies reported the existence of a similar microglia population in an AD model that localizes adjacent to Aβ plaques and expresses elevated levels of genes involved in suppressing inflammatory pathways (79). Ablation of this microglial subset results in an increased Aβ plaque load in multiple AD models, while an increase in this population is associated with Aβ plaque load reduction (80). Further, TREM2^−/−^ mice exhibit accelerated AD pathology coinciding with decreased microglial activation (81). Although these data suggest that DAM are a regulatory population, recent reports propose that DAM may be further subdivided into pro-inflammatory and anti-inflammatory subsets (82, 83). Proteins related to pro-inflammatory DAM are positively associated with neuropathology and precede cognitive decline (83). Moreover, heterogeneity may extend beyond this pro- and anti-inflammatory distinction (83). The vast majority of DAM studies center on AD, ALS, and MS (18). However, to date, no studies have examined DAM in NP-SLE. We find that microglia from both CReCOM and B6.*Sle1Sle3* models of NP-SLE are enriched for genes associated with DAM. Further, expression of these DAM-associated genes also correlates with the severity of behavioral deficits in CReCOM mice. However, as DAM themselves may be heterogeneous, characterizing microglia subsets, including DAM, in multiple NP-SLE models merits further investigation.

Here we show that the SLE-prone CReCOM model also exhibits a NP-SLE-like disease that mimics many of the pathologies observed in patients. Microglia are now at the forefront of investigations into neurological disorders and are implicated as key players in conditions ranging from neurodevelopmental (autism) to neurodegenerative (AD and chronic pain) disorders (84). However, very little is known regarding their role in NP-SLE. We have uncovered a novel 18-gene microglia signature common to both CReCOM and B6.*Sle1Sle3* strains. This “NP-SLE signature” suggests a more anti-inflammatory, regulatory role for NP-SLE microglia, potentially owing to an enrichment of the DAM subset in NP-SLE-like disease. Both “NP-SLE” and “DAM” signatures correlate with the severity of behavioral deficits in young SLE-prone mice. Thus, the discovery of our novel “NP-SLE signature,” as well as enrichment of the “DAM signature,” represents the first to connect microglia-specific expression patterns with clinical outcomes in NP-SLE-like disease prior to overt SLE-like disease. Further these data suggest that microglia-intrinsic defects precede contributions from peripheral SLE disease, and this link between associated signaling pathways in microglia and the onset of cognitive decline suggests a new area of investigation for NP-SLE. Macrophages transplanted into a new tissue environment may undergo epigenetic reprogramming to behave like macrophages already present within that tissue (85). Further, under microglia-depleting conditions, Ly6C^hi^CD62L^+^ classical monocytes can repopulate the compromised microglia niche and adopt key microglia-specific genes following engraftment in the brain (86). These data suggest a reprogramming of epigenetic and transcriptional profiles of classical monocytes to resemble that of brain-resident microglia. Since NP-SLE patients have decreased vascular integrity within the brain (2, 87), and classical monocytes may adopt a microglia-like phenotype, it could be argued that classical monocytes enter the brain to become microglia should the tissue-resident microglia population be compromised due to disease. Indeed, in multiple neurodegenerative diseases (AD and ALS) (88, 89), increased numbers of microglia have been observed in the parenchyma, and our own data suggest an expansion of the microglia population in brains of SLE-prone mice. Further, a microglia-like population was recently identified in human CSF that transcriptionally resembles DAM (90). Future studies will involve investigating the penetrance of our “NP-SLE” and “DAM” signatures in circulating monocyte subsets, as well as CSF-resident microglia-like cells of NP-SLE patients, and potentially correlating gene expression with the presence of soluble factors within the CSF. Development of these microglia-specific signatures and/or soluble factors within the CSF into indicative and/or predictive tools of NP-SLE in relatively easily obtainable patient samples would fulfill a critical need, as clinically validated biomarkers for diagnosis are currently non-existent and attributing NP-SLE manifestations to SLE in patients remains a challenge.

## Materials and Methods

### Mice

Mice homozygous for loxP flanked Caspase8 allele [*Casp8*^flox/flox^ (WT); generated on 129 background, backcrossed to C57BL/6 (B6) for at least 12 generations (91, 92)] were crossed with mice expressing Cre under control of the murine CD11c promoter (*CD11c*^cre^; generated on B6 background, Jackson 007567) to generate *CD11c*^cre^*Casp8*^flox/flox^ (CReCOM: Caspase-8 Removed CD11c-specific Overactive MyD88) mice as previously described (14). MRL^lpr/lpr^ mice (Jackson 000485) were utilized as positive controls and validated NP-SLE model. SLE-like disease in CReCOM mice was initiated by deletion of caspase-8 in peripheral CD11c^+^ conventional dendritic cells (14). We also observed caspase-8 deletion in tissue resident macrophage populations of the spleen and joint of CReCOM mice (14, 93). Thus, we examined caspase-8 deletion at the DNA level of brain infiltrating macrophages and microglia. While we did not observe caspase-8 deletion in brain infiltrating macrophages, microglia showed deletion of caspase-8 in CReCOM mice (Supplemental Figure 10A). Further, we confirmed deletion of caspase-8 in microglia, and not infiltrating macrophages, at the RNA level by RNA-seq (Supplemental Figure 10B). Mice were housed in barrier/specific-pathogen-free facilities at Northwestern University Center for Comparative Medicine (Chicago, IL, USA). Female mice were used in all studies. Transnetyx (Memphis, TN) performed genotyping and cell-specific caspase-8 gene deletion analysis. All procedures were approved by Northwestern University IACUC.

### Behavioral Assessment

Tests validated by Northwestern University Behavioral Phenotyping Core were performed to assess spatial memory, associative learning, acoustic startle response, motor learning/balance, anxiety, working memory, general activity, and gait. Age-matched 3–4-month-old MRL^lpr/lpr^, WT, and CReCOM mice and 9–10-month-old WT and CReCOM mice were housed in the core's animal holding facility. Instrument calibration was performed at the same time daily by the same individuals. Mice were equilibrated for 1 h prior to behavioral examination under low incandescent light.

#### Morris Water Maze

Spatial memory was assessed using the Morris water maze test (94, 95). This apparatus consisted of an open circular pool ~1.2 m in diameter, an array of visual navigational cues surrounding the pool area, an escape platform (hidden just under the surface of the water) placed in the middle of the pool, and an overhead camera connected to a computer running the image tracking software. This task took advantage of the natural swimming ability of rodents and tests for spatial memory. Animals were individually placed in a pool of water and allowed to swim until the mouse climbed up onto the target platform or after 60 s of search time. At the end of each trial (mice not finding the platform were guided to it), mice were allowed to rest on the platform for 10 s, reinforcing the associative pairing of location-reward. Mice were then dried off, returned to their home cage for 30 s and tested again before being returned to their cages to rest prior the next round of testing. Water within the pool was kept at 24–26°C and was made opaque with non-toxic white paint. Testing was performed at the same time each day by the same individuals. On the first day of testing, the mice were taught to climb onto the visible platform from the water and remained on the platform for 10 s prior to being returned to their home cage. From the second through the last day of testing (day 5), the platform was submerged 0.75 cm below the waterline, and remained in the same location for all testing while the starting locations changed. Parameters measured using the tracking software included path length and latency to platform. The three double-search trials starting from different locations were spaced 15–20 min apart: a span well-outside the range of working memory for mice, and requiring the hippocampus for recall and learning. The animal must learn to use visual cues around the maze to navigate a direct path to the hidden platform when started from different locations around the perimeter of the pool. Thus, decreased path length and latency to platform indicates increased spatial learning and memory over time. Animals with a deficit in spatial learning will find it hard to accomplish this task and will have increased latency to platform and distance traveled during the allotted timeframe.

#### Fear Conditioning

Associative learning was assessed using the fear conditioning test. This apparatus consisted of a training environment and a test environment. The conditioning environment was made up of a Plexiglas chamber (35 × 20 × 20 cm) with a metal grid electrifiable floor spaced 9 mm apart, placed inside a sound proof chamber with a light source and a speaker to transmit tone. The grids were wired to a computer-controlled activity monitor and tone/shock generator. Each sound proof chamber was evenly illuminated and equipped with a ceiling-mounted camera connected to a computer to monitor and capture the response of the mouse. The procedure was controlled by the FreezeFrame 4 software and analyzed with FreezeView software (Actimetrics, Wilmette, IL.). Before each test began, the walls, floors, and grids were cleaned with 70% ethanol to eliminate any olfactory interference. Each conditioning trial was a tone-pulse-shock method. This protocol consisted of a period of white noise, then a 75 dB, 10 kHz tone followed by a pulse, then a brief, mild (0.7 mA) foot shock through the metal grid floor. Approximately 24 h after the conditioning, the animal was returned to the same conditioning chamber without the tone-pulse-shock and its behavior was monitored to check for contextual conditioning. An absence or reduction of movement in this chamber indicates fear in response to the context of the conditioning chamber. Three hours after the test for contextual associative learning, the animals were returned to a different chamber (new visual surroundings, new odor) to test for cue-signaled fear independent of contextual conditioning. This was done by presenting the original conditioning tone in the novel context. A conditioned mouse exhibits fear by freezing in response to the conditioning context or cue. A failure to respond to either the conditioning context or the adverse stimuli (cue) by not freezing indicates a defect or malfunction in the amygdala and hippocampus.

#### Prepulse Inhibition

The acoustic startle response (ASR) to stimulus was assessed in the animals using the test for Prepulse inhibition (PPI) as previously described (96). Briefly the animals were placed in a sound attenuated chamber with tension plate floors to measure the response of the animals upon delivery of stimuli. A weak acoustic stimulus [the prepulse (S1)] was delivered first, and following a brief 100 ms interval, the stronger stimulus [pulse (S2)] was delivered. Then the startle amplitude was measured. The prepulses were 70 dB tones at three frequencies (4, 12, and 20 kHz). While 4 KHz is relatively low frequency for mice to detect, 20 KHz is the highest for the equipment and utilized for evaluating hearing loss is many mouse strains. Twelve KHz represents an optimal frequency for many mouse strains to determine alterations in prepulse inhibition (96). The pulses were delivered at 100 dB at the same frequencies. After 40 trials of the prepulse/pulse test, another 15 trials of just the pulse was run at 70, 80, 90, and 100 dB to establish a baseline of the acoustic startle response when there is no prepulse. The experiment was controlled and data were collected using Startle Reflex software (Med Associates, Inc., St. Albans, VT) and data analysis was performed using Microsoft Excel. The ratio of PPI is calculated as: startle amplitude of S1 & S2 paired stimulus, divided by the baseline S2 amplitude, ^*^100. A lower ratio means less startle, thus indicating that the delivery of the prepulse inhibits the startle response of the animal to the pulse. A reduction or inhibition of startle response as indicated by a lower PPI percentage reflects the ability of the CNS to temporarily adapt to a strong sensory stimulus when it is preceded by a weaker stimulus. Thus, an inability to adapt to the stronger signal, regardless of the prepulse, is represented by a higher PPI percentage indicating an increased startle response.

#### Rotarod

Acquisition of skilled behavior and motor coordination/balance in mice was assessed using the TSE RotaRod System, a machine comprising a rotating drum with individual lanes for each animal. Animal falls from the drum were detected by tension plate floors in each lane, and each lane was timed independently. Motor speed for drum rotation was controlled electronically. After training at constant speed (12 rpm, 60 s, 4 times/day) for 3 days, the speed of the rod was accelerated from 4 to 40 rpm. Four acceleration trials were performed with 20 min inter-trial intervals and the time until they dropped from the rod was recorded. The Rod software was used to define, store, and run speed profiles. Increased time before the fall and speeds at the fall indicate acquisition of motor learning/balance.

#### Zero Maze

Anxiety was assessed using the zero maze test. The zero maze was a round track (56 cm diameter) divided into four equal (two open and two closed) sections placed on an elevated stand and this task is based on the preference of mice for enclosed spaces. The closed sections are made up of two sets of walls along the track. The mice were placed on the track in the center of the open area and were examined for a preference for the closed or open arms. Each sound proof chamber was evenly illuminated and equipped with a ceiling-mounted camera connected to a computer to monitor and capture the response of the mouse. LimeLight software (Actimetrics, Wilmette, IL) was used to collect data for 300 s. Data were calculated as the percent of time in the open portions of the track. While an anxious mouse will avoid the open regions of the track, animals with a reduction in anxiety levels due to a neurological defect will spend an increased amount of time in the open space of the maze.

#### Y Maze

Immediate working memory performance was assessed on animals by recording spontaneous alternation behavior in a Y maze. The maze had three arms that radiated from a central triangular area and was placed in a sound proof chamber. The arms were spaced 120 degrees apart and were of identical dimensions. Each of the three arms was 60 cm in length, 3.5 cm wide at the bottom, and 14 cm wide at the top. A mouse was placed within the “home” arm and positioned with its nose toward the center of the maze and then released to choose one of the other arms. Each sound proof chamber was evenly illuminated and equipped with a ceiling-mounted camera connected to a computer to monitor and capture the response of the mouse. LimeLight software (Actimetrics) was used to collect data for 500 s while the mouse freely traversed the maze. The order of arm entries was recorded and analyzed for spontaneous alternation. An alternation was scored for each set of three consecutive choices where no repeated entries occurred. % alternation (# alternations/# of possible alternations^*^100) of 50% indicates a random selection. While young, healthy B6 mice typically exhibit scores of 75–80% alternation indicating acquisition of working memory, aging mice approach 50% alternation (97). The hippocampus governs spatial memory which will allow the rodents to alternate between the arms of the maze multiple times correctly. Animals with impaired working memory will repeatedly navigate the same arms of the maze instead of alternating between arms.

#### Open Field

The open field arena (56 × 56 cm) was used to assess levels of anxiety in the mouse as well as ambulation and activity levels as well as exploratory habits. Animals were placed in the center of the arena inside a sound proof chamber. Each sound proof chamber was evenly illuminated and equipped with a ceiling-mounted camera connected to a computer to monitor and capture the response of the mouse. LimeLight software (Actimetrics, Wilmette, IL) was used to collect ambulation activity for 300 s while the mouse freely traversed the arena. Data were calculated as the total distance traveled as well as the number of crossings in the arena as defined by the software. A hyperactive mouse will explore the test chamber and travel greater distances while an anxious mouse will spend more of its time along the perimeter and will not cross the arena.

#### Gait Analysis

Animals were tested on the DigiGait™ Imaging System (Mouse Specifics Inc., Framingham, MA). This is a widely used ventral plane videography instrumentation for gait analysis. The ventral images of the animal were captured by a video camera anchored below a transparent treadmill belt. One animal is placed in the arena at a time. Data were collected at 10, 17, and 24 cm/s for two trials at each speed. The Digigait™ arena and belt was cleaned with 70% ethanol in between animals. Footprints from a segment of a 4–5 s video was analyzed using Digigait™ Analysis version 15. Maximum speed before stumbling was noted, and gait symmetry at all three speeds was calculated. A gait symmetry score of 1.0 indicates symmetrical ambulation and no defect in coordination or locomotion.

### MRI Acquisition and Processing

Each mouse was scanned using a 7T Clinscan MRI (Bruker, Billerica, MA) using clinical grade software platform (Syngo, Siemens). Twenty minutes prior to scanning, a tail-vein catheter was inserted and primed with MR gadolinium-based contrast agent (~0.3 mmol/Kg body weight; Prohance, Bracco). Mice were anesthetized (isoflurane mixed with 100% O_2_) and placed in a dedicated holder with stereotactic head alignment. Respiration and body temperature were monitored with dedicated physiological monitoring system (SAI, New Jersey, USA) with anesthesia continuously delivered through a nose cone. Using a four-channel mouse brain surface coil placed on mouse's head, the following were used to acquire images: (1) localizer tri-axial gradient echo sequence for positioning; (2) T1-weighted gradient echo 3D sequence with TR = 70 ms and TE = 1.6 ms and isotropic spatial resolution 150 μm; (3) dynamic T1-weighted GRE series of images (120 repetitions) with 3.6 s temporal resolution used for acquisition of dynamic contrast enhanced image series (DCE-MRI) by injecting the agent at ~20th image set; (4) multi-direction (64) and multiple value diffusion gradient (0, 200, 800) 2D EPI sequence TR = 2,000 ms and TE = 25 ms with in-plane resolution of 200 μm and 12 longitudinal slices covering whole brain. Each scanning session lasted ~20–30 min. Anatomical and morphological analysis was conducted using 3D brain images acquired at 150 μm isotropic resolution. Classification criteria for lesion detection using ITK-SNAP (http://www.itksnap.org) (98) assumed that lesions would exhibit a higher signal intensity compared to signals from normal surrounding brain tissue (~30% higher for lesion; ~30–50% higher for ventricle/CSF). Fractional anisotropy (FA) maps were generated using DTI processing packages contained in the image acquisition and processing platform (Syngo, Siemens) from diffusion MRI images. To facilitate detection/quantification of FA differences across groups using multi-slice dataset, we used a similar threshold approach to above. Using 2D FA maps and ROI tool in JIM 7.0 (Xinapse, Essex, UK), high FA areas were segmented in each slice to generate whole brain 3D-rendered FA surfaces. Total volumes were extracted. FA reduction using 3D volumetric data reflects loss of white matter. 3D FA-rendered surfaces enabled visualization of abnormal connectivity patterns reflected in morphometric alterations. DCE-MRI data was analyzed using least-squared fitting algorithm and standard two-compartment pharmacokinetic model (Tofts). Ktrans maps were generated and considered to reflect vascular integrity.

### Tissue Processing, Flow Cytometry, and Fluorescence Activated Cell Sorting (FACS)

Brain tissue processing, flow cytometric analysis, and FACS-purification of macrophages and microglia were performed following behavioral task completion (testing lasted ~6 weeks), as previously described by us (17, 47). Briefly, mice were transcardially perfused with ice cold HBSS. Brains were excised, meninges were removed, and brains were placed in ice cold HBSS until processing. Brains were weighed, infused with digestion buffer [2.5 mg/mL Liberase TL (Roche, Basel, Switzerland) and 1 mg/mL DNase I in HBSS] using a 30 g needle, cut into small pieces, and placed into C-tubes (Miltenyi Biotec) containing 4 mL digestion buffer. C-tubes were placed on GentleMACS dissociator (m_brain_3_protocol; Miltenyi Biotec), incubated (30 min, 37°C, 200 rpm shaking), and placed on GentleMACS dissociator. Released cells were passed through 40 μm nylon mesh using a cell masher and washed with 100 ml autoMACS Running Buffer (Miltenyi Biotec). Microglia and infiltrating cells were isolated using 30/70-percoll gradient (Percoll Plus, GE Healthcare). Cells collected from gradient interphase were washed with HBSS and counted (Countess, Invitrogen); trypan blue discriminated dead cells. Cells were stained with live/dead Aqua viability dye (Invitrogen), incubated with Fc-Block (BD Biosciences), and stained with fluorochrome-conjugated antibodies (Supplemental Figure 11). Siglec H expression confirmed gating strategy to discriminate between microglia and macrophages (Supplemental Figure 10C), as microglia, not macrophages, express this marker (28). We did not detect monocyte-derived dendritic cells (DCs) in brain, as CD11b^+^CD11c^+^ cells expressed CD64 (Figure 3A), and DCs do not express CD64 (99). For analysis of intracellular synaptic vesicle glycoprotein 2A (SV2A), a prototypic protein identified in synaptic vesicles of neurons and endocrine granules that regulates action potential-dependent neurotransmitter release (100–103), cells were permeabilized (BD Cytofix/Cytoperm Fixation/Permeabilization Kit) according to manufacturer's instructions, and data were acquired on BD LSR2 (BD Biosciences, San Jose, CA). For sorting experiments, data were acquired on BD FACSAria cell sorter (BD Biosciences) at the Northwestern University RHLCCC Flow Cytometry Facility and populations were sorted. Pelleted sorted cells were immediately lysed in extraction buffer from PicoPure RNA isolation kits (Arcturus Bioscience, Inc.). Lysates were stored at −80°C. Flow cytometric data analysis was performed using Flowjo (TreeStar, Ashland, OR).

### RNA Sequencing (RNA-Seq)

RNA-seq was performed as previously described by us (17, 47) on mice that underwent behavioral testing. Briefly, RNA from FACS-purified macrophages and microglia of age-matched 5–6-month-old and 11–12-month-old female WT and CReCOM mice were extracted using PicoPure RNA isolation kits according to manufacturer's instructions. Sample quality control, processing, and library preparation were performed by the Northwestern University Next Generation Sequencing Core. RNA quality/quantity were measured using Agilent High Sensitivity RNA ScreenTape System (Agilent Technologies). DNA libraries were prepared from total RNA from macrophages (1 ng) and microglia (2 ng) using a QuantSeq 3′ biased mRNA-seq Library Prep Kit for Illumina (Lexogen Inc.) and sequenced on Illumina NextSeq 500 instrument (Illumina Inc.) with ~10 million reads/sample target read depths. Raw sequencing files were de-multiplexed using bcl2fastq. Fastq files were trimmed of low-quality reads and bases, polyA tails, and adaptors using bbduk (http://jgi.doe.gov/data-and-tools/bb-tools/). Trimmed fastq files were aligned to mouse reference genome (mm10,GRCm38) using STAR algorithm (104). HTSeq on resulting BAM files provided raw gene counts, which were merged into a single table and normalized for read depth using counts per million (CPM). For macrophages and microglia, three and four samples, respectively, from each group were included for subsequent analyses. Samples were excluded based on failure of mice to respond to behavioral testing, issues with brain processing (poor perfusion, sorter error), systemic disease (spleen and cervical lymph node weights), insufficient RNA, read count <7,000,000, low alignment (<56% for macrophages; <70% for microglia), and/or high percentage unmapped reads (too short; >28% for macrophages; >20% for microglia) (Supplemental Figures 12–15). Libraries showed an average of 66% alignment for macrophages and 80% alignment for microglia. For analysis, we focused on highly expressed genes, defined as “log_2_(maximum mean CPM of any experimental group+1)>5” for macrophages (5642) and “log_2_(maximum mean CPM of any experimental group+1)>4” for microglia (7771). DESeq2 defined differential genes. GENE-E (https://software.broadinstitute.org/GENE-E/) generated correlation matrix and heat maps. Ggplot2 package in RStudio (RStudio Inc.) generated plots. GOrilla defined GO associations and related *p*-values (105). A volcano plot was generated using the fold change of normalized gene counts (log2[CPM+1]) between 11-12-month-old WT and CReCOM macrophages on x-axis and *p*-values (–log_10_) on y-axis. A heat map was generated using fold change of normalized gene counts (log_2_[CPM+1]) between 11-12-month-old WT and CReCOM microglia. For comparative analyses between NP-SLE microglia, we focused on aforementioned highly expressed genes for aged CReCOM microglia (7771) and the previously published dataset for 8–10-month-old B6.*Sle1Sle3* microglia (8151) (17). Hypergeometric distribution tests determined significance of overlap between upregulated genes in CReCOM and B6.*Sle1Sle3* microglia compared to respective controls (7076 common gene background). Hypergeometric distribution tests were also performed between NP-SLE microglia and DAM (29) [common gene background for comparison of DAM to: CReCOM microglia (7060), B6.*Sle1Sle3* microglia (7291), CReCOM macrophages (5204)] as well as “NP-SLE” and “DAM” signatures (6,486 common gene background). For scatter plots, behavior and gene expression scores (“NP-SLE” and “DAM” signatures) were generated for each animal. Animals were ranked 1–8 per age group (1 = normal behavior; 8 = abnormal behavior) for disease-associated behavioral tests to obtain an average ranking for Morris water maze (latency on days 2–5), Fear Conditioning (% freezing for context and cue), PPI (% PPI at 4, 12, and 20 kHz), and Rotarod (time before drop on days 4–5). Rankings were added together to obtain behavior scores (4–32). For “NP-SLE” and “DAM” signatures, expression was normalized for each gene to obtain a value from 0 to 1, and normalized gene expression values were added together to obtain gene expression scores.

### Statistical Analysis

Investigators were not blinded to treatment groups during behavioral testing. GraphPad Prism 7.0 was used for statistical analyses. Comparisons were made using non-paired, non-parametric Mann-Whitney tests, unless indicated. Behavioral and flow cytometric analysis experiments were repeated three times. For scatter plots, correlation coefficient values were determined by Pearson method of correlation. Data reported as mean ± SEM, unless indicated.

## Data Availability Statement

The datasets generated for this study can be found in Mendeley, at 10.17632/gphwtzz36y.1 and 10.17632/8j2gsn3bwv.2.

## Ethics Statement

The animal study was reviewed and approved by Northwestern University IACUC.

## Author Contributions

HM carried out all *in vivo* animal experiments, performed behavioral studies, analyzed RNA-seq data, and assisted with drafting the manuscript. DW guided RNA-seq studies, analyzed RNA-seq data, and assisted with drafting the manuscript. DP performed MRI on all animals, generated quantitative data and images, and revised the manuscript. EM assisted with analyzing RNA-seq data and revised the manuscript. AS assisted with *in vivo* animal experiments and revised the manuscript. MK assisted with performing behavioral data analysis and revised the manuscript. GG assisted with analyzing RNA-seq data and revised the manuscript. SD assisted with retinal angiography studies and revised the manuscript. CB, SD, and MM assisted with *in vivo* animal experiments and revised the manuscript. JL performed retinal angiography studies and revised the manuscript. CP participated in the conception, design, and coordination of the study, and revised the manuscript. CC participated in the conception, design, and coordination of the study, assisted with all *in vivo* animal experiments, assisted with behavioral testing, performed behavioral data analysis, assisted with analyzing RNA-seq data, performed all statistical analyses, and drafted the manuscript. All authors read and approved the manuscript.

### Conflict of Interest

The authors declare that the research was conducted in the absence of any commercial or financial relationships that could be construed as a potential conflict of interest.
