# Supplementary material for: A Novel Microglia-Specific Transcriptional Signature Correlates With Behavioral Deficits in Neuropsychiatric Lupus
Source: Front Immunol. 2020 Feb 26;11:230. doi: 10.3389/fimmu.2020.00230 (PMC7055359; doi:10.3389/fimmu.2020.00230)
Supplement: Supplementary file 1 [file Data_Sheet_1.pdf]

Supplementary Material

1 Supplementary Materials and Methods

Fluorescein Angiography.

Mice were anesthetized via intraperitoneal delivery of ketamine (90 mg/kg, Akron, Lake Forest, IL)/xylazine (12 mg/kg, Akorn) cocktail. Eyes were anesthetized with 1 drop of 0.5% tetracaine (Alcon, Fort Worth, TX). Pupils were dilated with 1 drop of 2.5% phenylephrine (Akorn) and 0.5% tropicamide (Akorn). Whiskers were trimmed with scissors. A subcutaneous injection of meloxicam (1 mg/kg, Henry Schein Animal Health, Melville, NY) was given for pain control. Fluorescein (0.03-0.05 ml, 100 mg/ml, Akorn) was injected into the tail vein. The fundus was imaged using the Micron3 fundus camera (Phoenix Technology Group, Pleasanton, CA). After imaging, mice given ophthalmic ointment (Akorn) and placed in a heated recovery cage.

Histopathology and Immunohistochemistry.

Animals were sacrificed and perfused using 4% paraformaldehyde in PBS, brains were excised and fixed in 4% paraformaldehyde. Fixed brains were sent to the Northwestern University Mouse Histology & Phenotyping Laboratory for further processing. Briefly, paraffin-embedded brain sections (4 μm) were stained with anti-mouse CD45. All images were photographed at X2 and X10 using an Olympus BX41 microscope equipped with an Olympus DP21 camera. Slides were used for qualitative and not quantitative purposes and were thus not scored by a pathologist.

2 Supplementary Figures

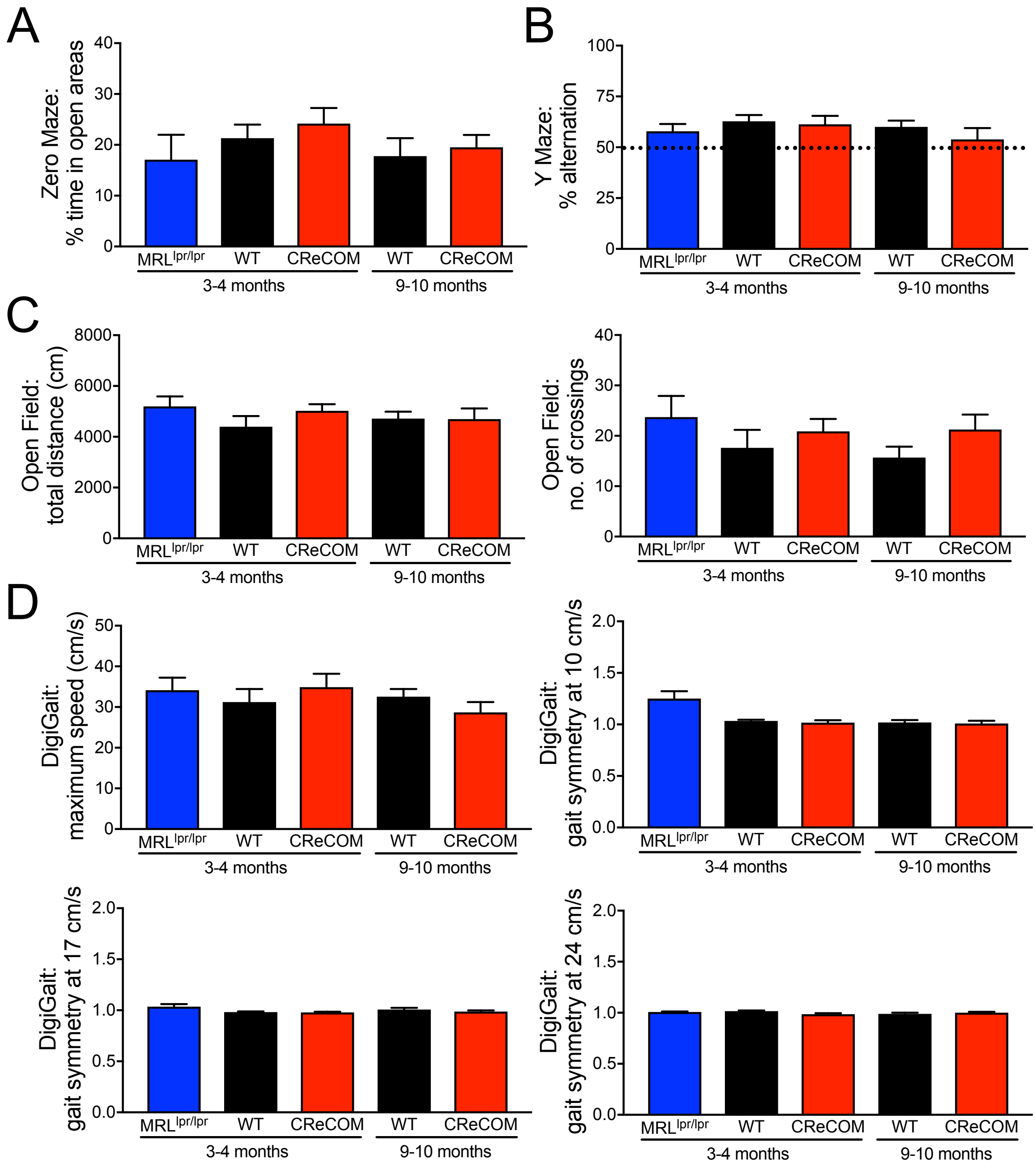

Supplemental Figure 1. CReCOM mice exhibit similar anxiety levels, alternation, activity levels and gait to WT mice. 3-4-month-old female MRL$^{lpr/lpr}$ (n=7), WT (n=9), and CReCOM (n=8) and 9-10-month-old WT (n=9) and CReCOM (n=9) mice underwent behavioral testing. Data are combined from 3 independent experiments. (**A**) Zero maze: % time spent in open areas. (**B**) Y maze: % alternation. (**C**) Open field: total distance travelled and number of crossing across the arena. (**D**) DigiGait: maximum speed that the animals could maintain and gait symmetry at 10, 17 and 24 cm/s.

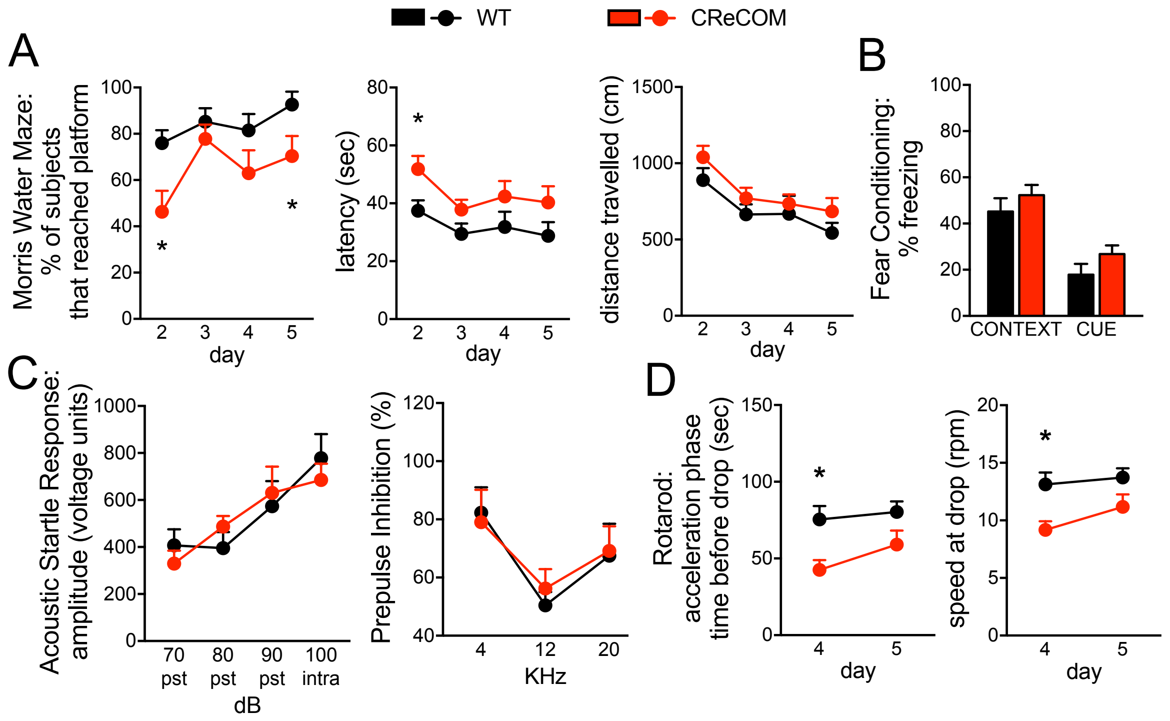

Supplemental Figure 2. Behavioral deficits persist in aged CReCOM mice. 9-10-month-old female WT (n=9) and CReCOM (n=9) mice underwent behavioral testing. Data are combined from 3 independent experiments. (**A**) Morris water maze: % of mice that reached the hidden platform, latency to platform and distance travelled. (**B**) Fear conditioning: % freezing following reintroduction of "shock" environment (CONTEXT) or tone associated with "shock" (CUE). (**C**) Prepulse inhibition: amplitude of acoustic startle response at 70, 80, 90, and 100 dB and % prepulse inhibition at 4, 12, and 20 KHz frequencies. (**D**) Rotarod: time spent on rod prior to drop and speed at drop during acceleration phase. (*,$p<0.05$).

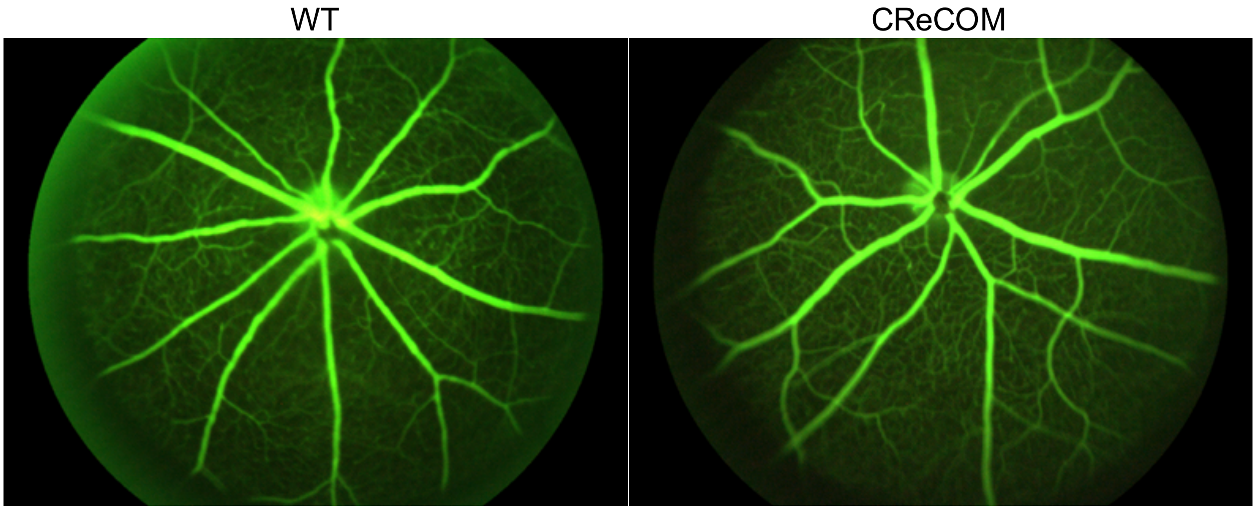

Supplemental Figure 3. CReCOM mice do not exhibit vascular ischemia or leakage in the retina. 11-12-month-old female WT (n=5) and CReCOM (n=5) mice underwent fluorescein angiography. Representative images are shown.

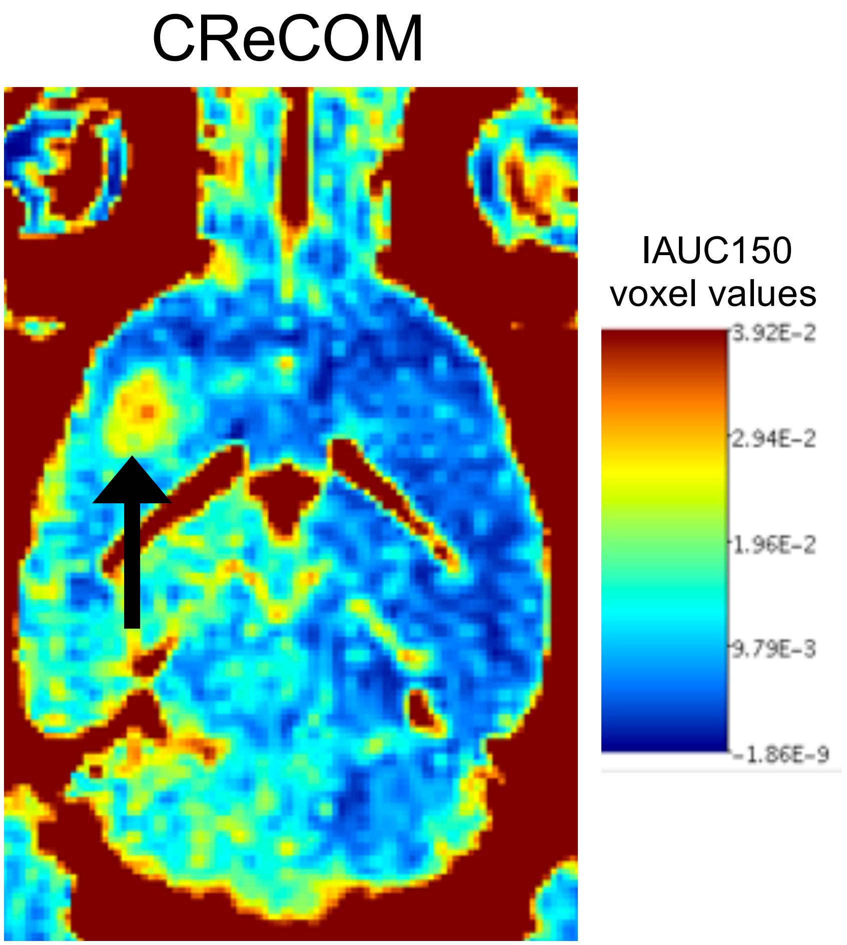

Supplemental Figure 4. Presence of a lesion in a CReCOM mouse. 4-month-old female MRL$^{lpr/lpr}$ (n=4) and 12-month-old WT (n=4) and CReCOM (n=4) mice underwent dynamic contrast-enhanced MRI. Shown is a representative IAUC150 map of one of the CReCOM mice. Black arrow indicates a large lesion.

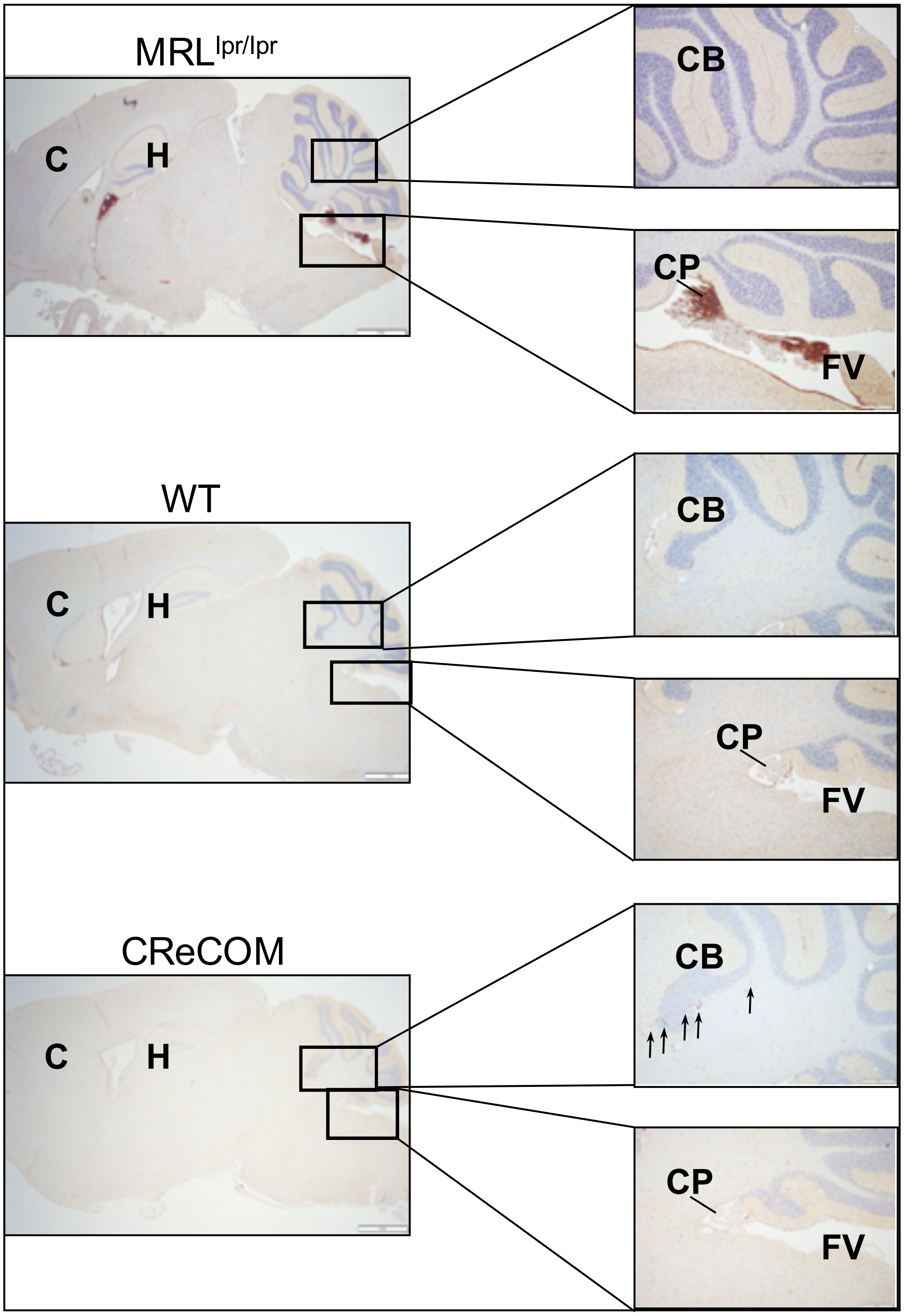

Supplemental Figure 5. Histological evidence of diffuse infiltration in CReCOM brain parenchyma, but not the choroid plexus. 4-month-old female $MRL^{lpr/lpr}$ (n=2) and 12-month-old WT (n=2) and CReCOM (n=2) mice were perfused with 4% paraformaldehyde in PBS. Brains were extracted, paraffin-embedded and sequentially sliced along the sagittal plane. Sections (4 µm) were stained with an antibody specific for mouse CD45. Images shown on the left were taken at a 20X magnification with an exposure time of 66.7 microsecond. Images on the right were taken at a 100X magnification with an exposure time of 156.3 microsecond. Black arrows denote CD45 positivity in the cerebellum. C-cortex; H-hippocampus; CB; cerebellum; CP-choroid plexus; FV-fourth ventricle.

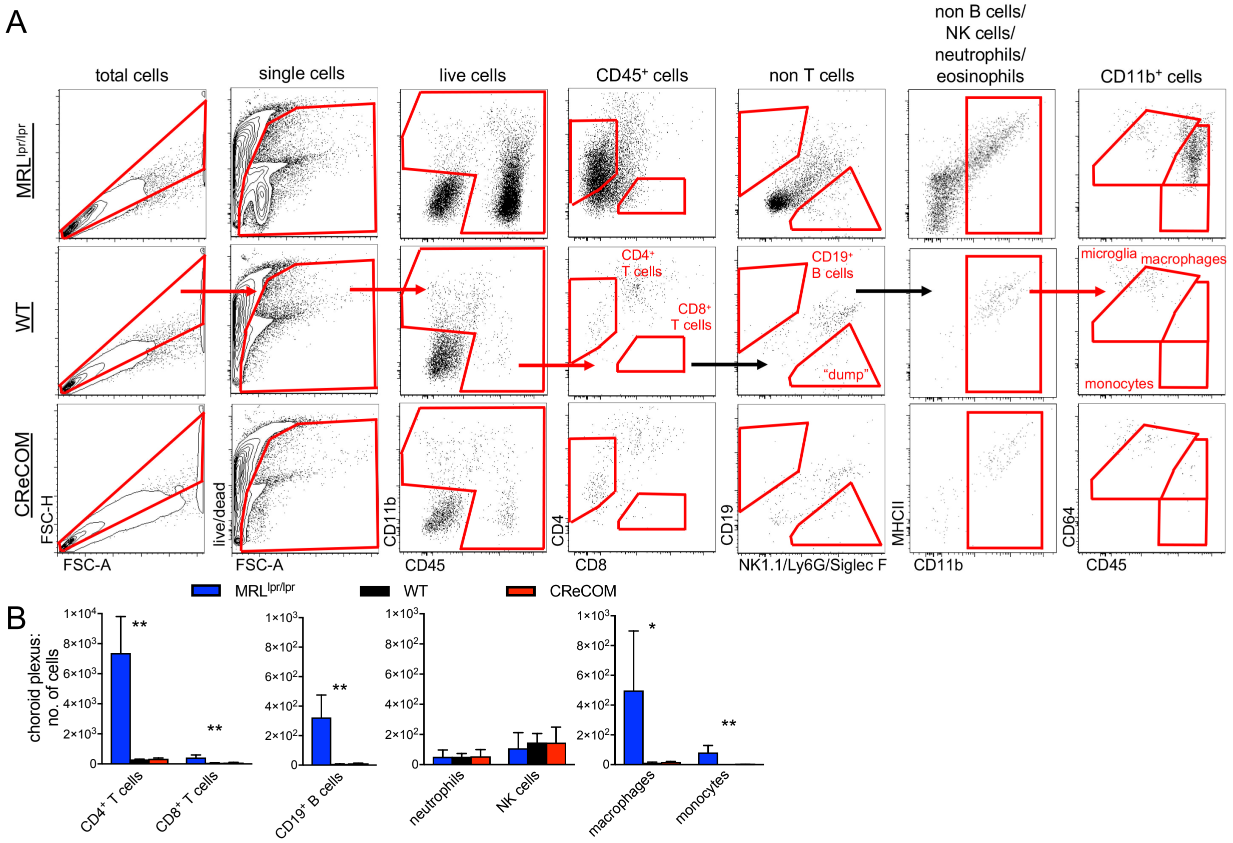

Supplemental Figure 6. CReCOM mice do not show evidence of choroid plexus infiltration. The choroid plexuses of 3-4-month-old female MRL$^{lpr/lpr}$ (n=5) and 9-11-month-old WT (n=7) and CReCOM (n=7) mice were isolated and analyzed by flow cytometry. Data are combined from 2 independent experiments. (**A**) Choroid plexus gating strategy. (**B**) Quantitative analysis of cell populations in the choroid plexus. (*,p<0.05; **,p<0.005).

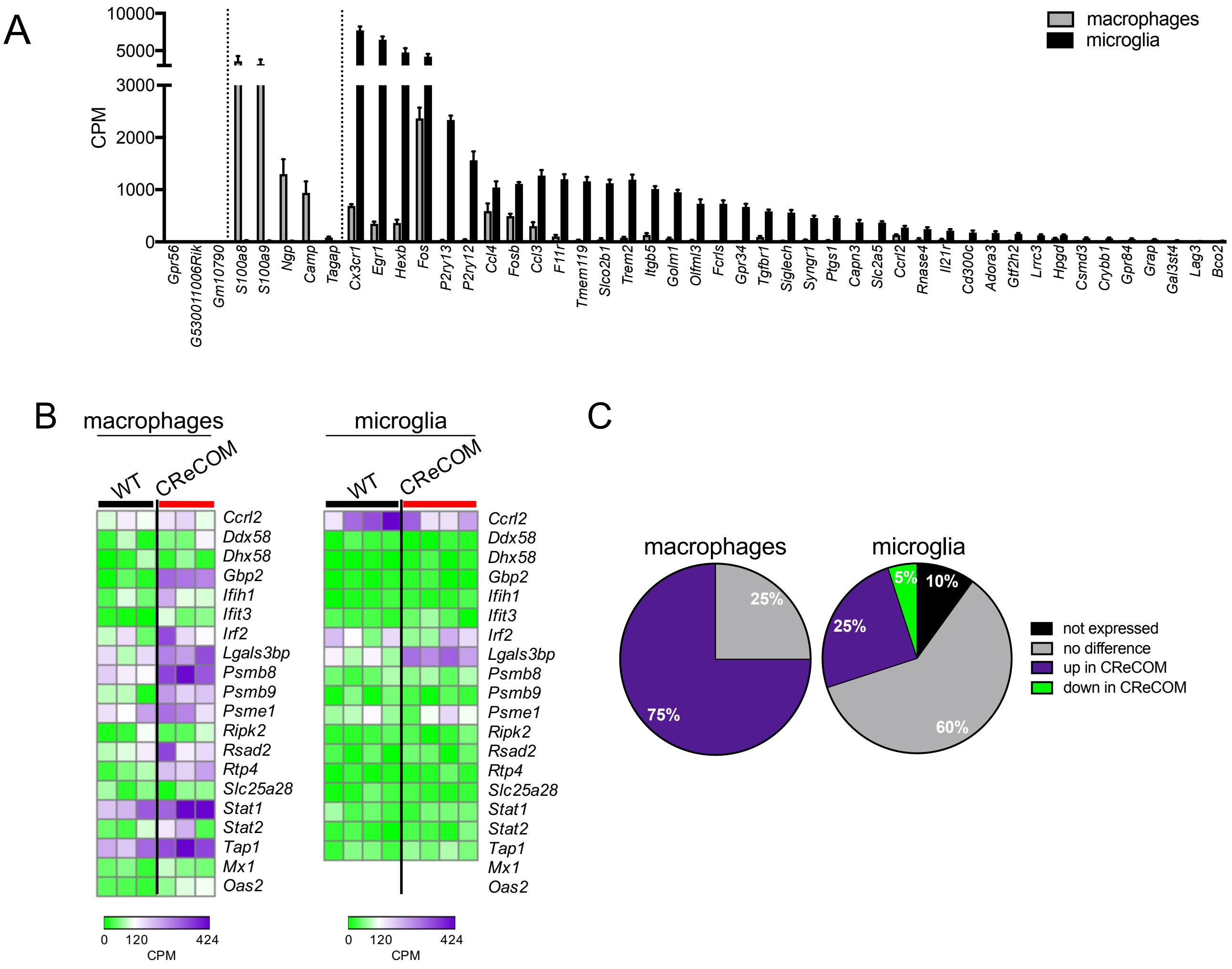

Supplemental Figure 7. Microglia express microglia-specific genes but do not exhibit an IFN signature. FACS-purified CD11b^{+}CD64^{+}CD45^{hi} macrophages of 11-12-month-old female WT (n=3) and CReCOM (n=3) mice and CD11b^{+}CD64^{+}CD45^{lo} microglia of 11-12-month-old female WT (n=4) and CReCOM (n=4) mice were analyzed by RNA-seq. (**A**) Expression values (counts per million=CPM) for 47 known microglial genes in macrophages and microglia. Values shown are combined from WT and CReCOM samples. (**B**) Heat maps of CPM values for 20 IFN response genes for both macrophages and microglia. (**C**) Pie charts allocating 20 IFN response genes into groups based on CReCOM expression level compared to WT.

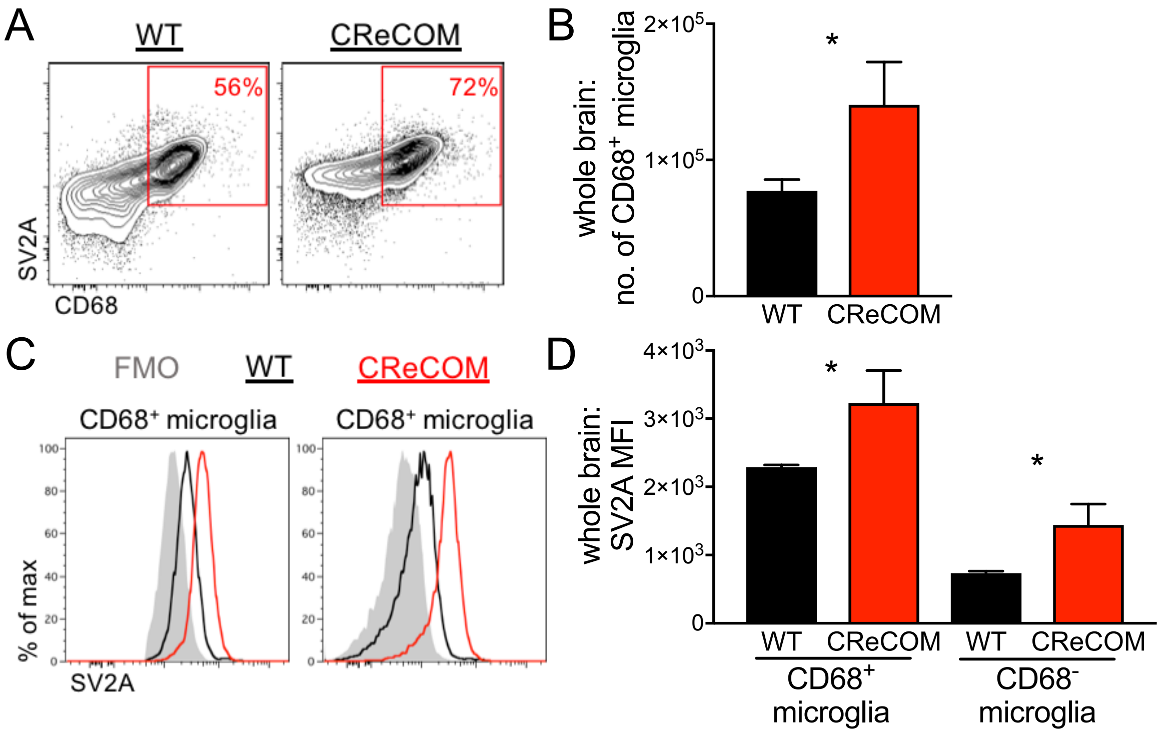

Supplemental Figure 8. CReCOM microglia are more activated and show increased uptake of synaptic material. Brains of 9-11-month-old female WT (n=3) and CReCOM (n=4) mice were analyzed by flow cytometry. $CD11b^+CD64^+CD45^{lo}$ microglia were gated on for subsequent analysis. Data are representative of 2 independent experiments. (**A**) Representative plots of SV2A and CD68 expression. (**B**) Quantitation of $CD68^+$ microglia. (**C**) Representative histogram of intracellular SV2A in $CD68^+$ and $CD68^-$ microglia. Fluorescence minus one (FMO) is presented as a negative control. (**D**) Quantitation of SV2A expression in $CD68^+$ and $CD68^-$ microglia.

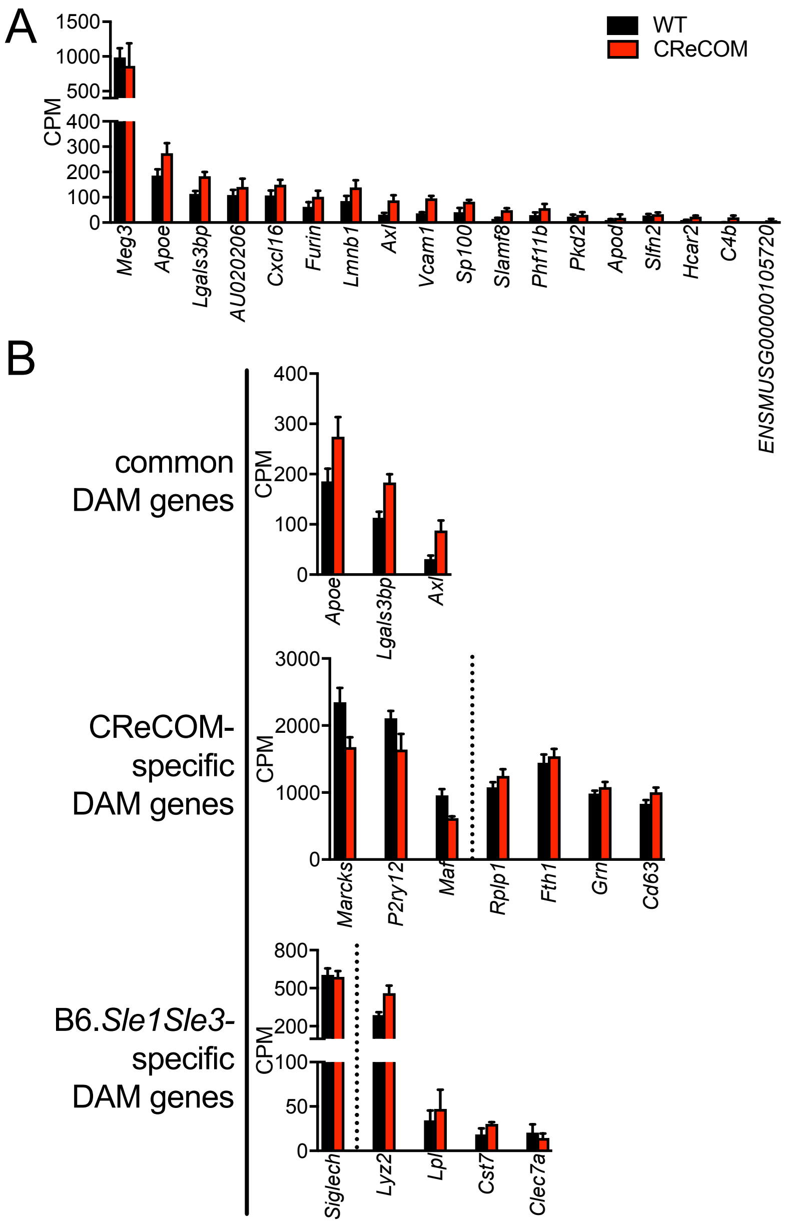

Supplemental Figure 9. Microglia from the 3-4-month-old cohort exhibit similar expression patterns of 'NP-SLE' and 'DAM' signatures as the 9-10-month-old cohort. FACS-purified $CD11b^{+}CD64^{+}CD45^{lo}$ microglia of 5-6-month-old female WT (n=4) and CReCOM (n=4) mice were analyzed by RNA-seq. Expression values (CPM) for (**A**) 'NP-SLE' and (**B**) 'DAM' signatures.

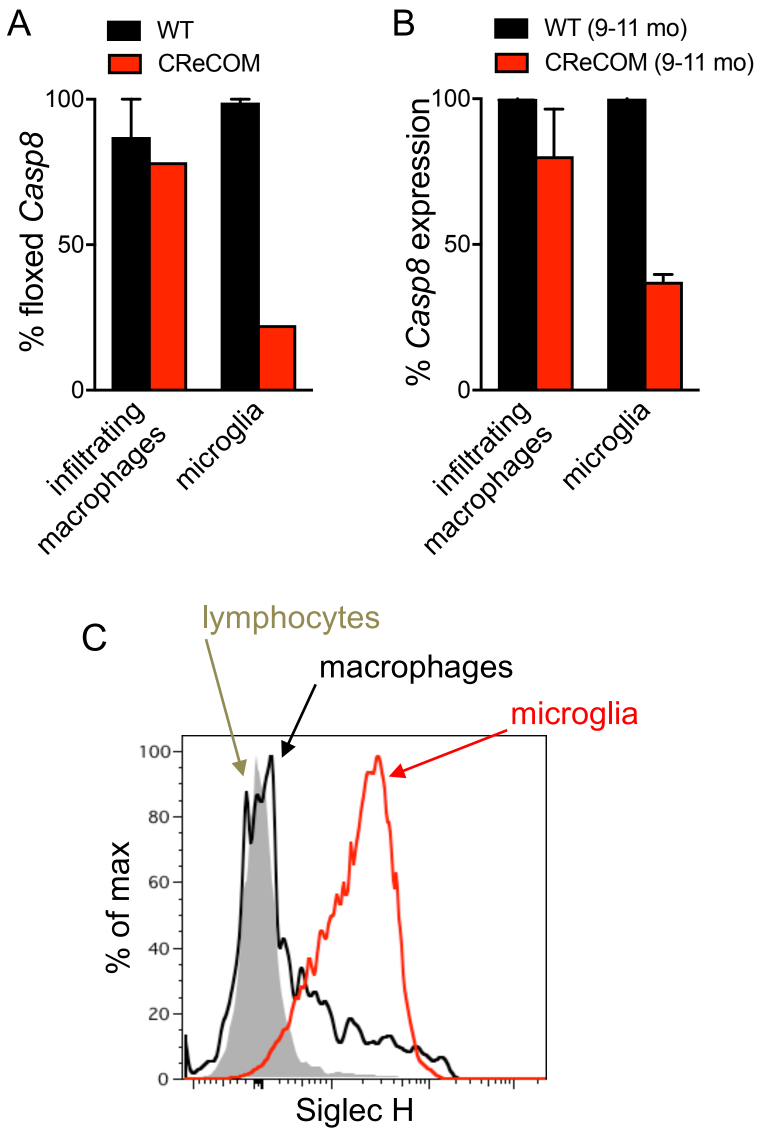

Supplemental Figure 10. Brain infiltrating macrophage and microglial expression of Siglec H and caspase-8. (**A**) $CD11b^+CD64^+CD45^{hi}$ macrophages and $CD11b^+CD64^+CD45^{lo}$ microglia were FACS-purified from 9-11-month-old female WT (n=2) and CReCOM (n=2) mice. DNA was analyzed for the presence of the caspase-8 floxed allele. Data are presented as % floxed and are derived as follows: divide the level of the floxed allele from a CReCOM-sorted population by the level of the floxed allele from the WT-sorted population and convert the resulting value into %. (**B**) FACS-purified $CD11b^+CD64^+CD45^{hi}$ macrophages of 11-12-month-old female WT (n=3) and CReCOM (n=3) mice and $CD11b^+CD64^+CD45^{lo}$ microglia of 11-12-month-old female WT (n=4) and CReCOM (n=4) mice were subjected to Quantseq 3' biased RNA-seq protocol. Data are presented as % *Casp8* expression and are derived as follows: divide the CPM from a CReCOM-sorted population by the CPM from the WT-sorted population and convert the resulting value into %. (**C**) Brains of 9-11-month-old female WT (n=4) and CReCOM (n=4) mice were analyzed by flow cytometry. Expression of Siglec H on $CD4^+CD8^+CD19^+$ lymphocytes (gray-filled), $CD11b^+CD64^+CD45^{hi}$ macrophages (black line), and $CD11b^+CD64^+CD45^{lo}$ microglia (red line).

Supplemental Figure 11. List of antibodies utilized for brain flow cytometric analysis.

Antigen	Clone	Fluorochrome	Manufacturer
CD45	30-F11	FITC	eBioscience
CD19	1D3	PE	BD Biosciences
MHC II	M5/114.15.2	PerCP-Cy5.5	Biolegend
CD68	FA-11	PerCP-Cy5.5	Biolegend
CD8	53-6.7	efluor 450	eBioscience
CD4	RM4-5	Alexa700	BD Biosciences
CD11b	M1/70	APC-Cy7	BD Biosciences
Siglec H	eBio440c	PE-Cy7	Invitrogen
Ly6G	1A8	PE-CF594	BD Biosciences
NK1.1	PK136	PE-CF594	BD Biosciences
Siglec F	E50-2440	PE-CF594	BD Biosciences
CD64	X54-5/7.1	APC	Biolegend
SV2A	polyclonal	Alexa647	Bioss

Supplemental Figure 12. Parameters for macrophage (11-12-month-old) sample exclusion.

Sample	*Spleen Weight (mg)*	*Cervical Lymph Node Weight (mg)*	*cDNA library (pg/mL)*	*# Untrimmed Reads*	*% Uniquely Mapped Reads (alignment)*	*% Unmapped: Too Short*	*Reason for Exclusion*
C8_O_F1_MAC	77.3	15.4	449	11,501,116	60.24%	23.56%	histogram
C8_O_F2_MAC	76.3	14.3	1450	14,556,822	66.50%	16.64%	
C8_O_F3_MAC	75.3	13					insufficient RNA
C8_O_F4_MAC	83.7	17.9	1680	9,289,974	69.00%	16.56%	
C8_O_F5_MAC	73.3	18.3					insufficient RNA
C8_O_F6_MAC	68.7	13.4	774	11,701,518	43.67%	45.05%	behavior/ sorter error/ 43.67% alignment/ histogram/ high % unmapped
C8_O_F7_MAC	99	15.5	278	11,012,854	63.89%	21.12%	
CD11cC8_O_F1_MAC	171.9	204.5	1740	18,224,423	66.72%	16.77%	
CD11cC8_O_F2_MAC	147.2	38.6	942	12,132,134	64.13%	19.32%	mouse (low spleen and cervical lymph node weights)/ histogram
CD11cC8_O_F3_MAC	380	301.4	2500	15,701,172	65.81%	19.08%	
CD11cC8_O_F4_MAC	223.7	168.1	741	10,807,502	64.63%	20.26%	
CD11cC8_O_F5_MAC	114	111.8	649	13,814,153	55.33%	28.83%	mouse (low spleen weight)/ 55.33% alignment/ histogram/ high % unmapped

Supplemental Figure 13. Parameters for microglia (5-6-month-old) sample exclusion.

Sample	*Spleen Weight (mg)*	*Cervical Lymph Node Weight (mg)*	*cDNA library (pg/mL)*	*# Untrimmed Reads*	*% Uniquely Mapped Reads (alignment)*	*% Unmapped: Too Short*	*Reason for Exclusion*
C8_Y_F1_MIC	70.5	10.1	1370	8913743	80.81%	5.85%	
C8_Y_F2_MIC	87.2	12	1030	7425062	79.56%	7.09%	
C8_Y_F3_MIC	58.9	11.7	218	11547611	75.64%	9.58%	low cDNA library
C8_Y_F4_MIC	89.4	20.2	871	9035937	73.94%	11.86%	behavior/histogram
C8_Y_F5_MIC	67.3	13.7	947	9727757	80.05%	6.96%	
C8_Y_F6_MIC	81.2	10.8	583	9057911	75.85%	10.83%	low cDNA library
C8_Y_F7_MIC	90.2	14.9	1640	10026388	79.15%	7.60%	
CD11cC8_Y_F1_MIC	227.5	106.4	72.1	5787773	64.70%	21.37%	sorter error/ low cDNA library/ low read count/ 64% alignment/ high % unmapped
CD11cC8_Y_F2_MIC	176.2	75.4	1260	10689194	74.43%	12.21%	
CD11cC8_Y_F3_MIC	126.8	37	1670	7789190	80.57%	5.67%	
CD11cC8_Y_F4_MIC	270.6	119.1	2130	10037007	79.00%	7.20%	
CD11cC8_Y_F5_MIC	222.1	100.1	1640	9505555	80.11%	6.14%	

Supplemental Figure 14. Parameters for microglia (11-12-month-old) sample exclusion.

Sample	*Spleen Weight (mg)*	*Cervical Lymph Node Weight (mg)*	*cDNA library (pg/mL)*	*# Untrimmed Reads*	*% Uniquely Mapped Reads (alignment)*	*% Unmapped: Too Short*	*Reason for Exclusion*
C8_O_F1_MIC	77.3	15.4	1150	9168109	80.83%	5.84%	
C8_O_F2_MIC	76.3	14.3	1810	10337191	80.21%	6.66%	
C8_O_F3_MIC	75.3	13	1150	9483528	80.11%	6.52%	
C8_O_F4_MIC	83.7	17.9	522	8872189	76.04%	10.79%	low cDNA library/ histogram
C8_O_F5_MIC	73.3	18.3	1640	7796155	79.50%	7.20%	
C8_O_F6_MIC	68.7	13.4	854	9740996	61.53%	27.08%	behavior/ sorter error/ 61% alignment/ high % unmapped
C8_O_F7_MIC	99	15.5	387	10432666	75.03%	11.19%	mouse (high spleen and cervical lymph node weights)/ low cDNA library
CD11cC8_O_F1_MIC	171.9	204.5	1620	8943289	79.76%	6.93%	
CD11cC8_O_F2_MIC	147.2	38.6	1400	8077132	80.03%	6.41%	mouse (low spleen and cervical lymph node weights)/ histogram
CD11cC8_O_F3_MIC	380	301.4	832	10015574	77.71%	8.78%	
CD11cC8_O_F4_MIC	223.7	168.1	696	11064475	78.94%	7.34%	
CD11cC8_O_F5_MIC	114	111.8	646	10642333	77.31%	8.74%	

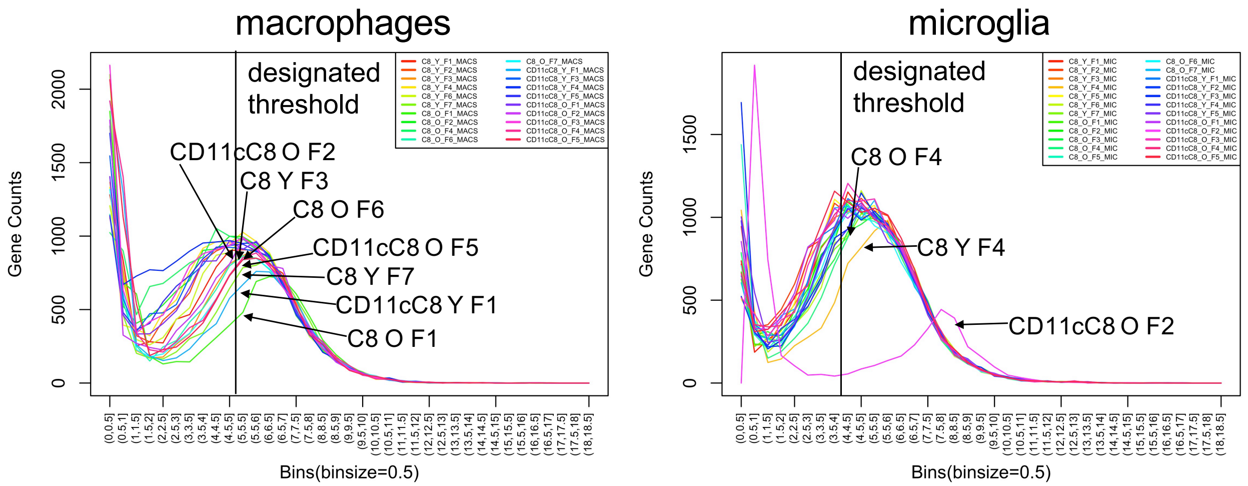

Supplemental Figure 15. Histogram for RNA-seq sample quality check. RNA was extracted from FACS-purified macrophages and microglia of 5-6-month-old and 11-12-month-old WT and CReCOM mice. Following RNA extraction, cDNA library preparation, and sequencing, demultiplexed reads were converted into CPM and frequencies of genes at incremental expression levels were determined and depicted in histogram form. Vertical black lines on frequency polygons indicate designated thresholds determined for minimum gene expression level in both populations.
